# Design and Development of Miniature Measuring Instrument for Parachute Cords Dynamic Load for Stepless Parachute Opening

**DOI:** 10.3390/s24196232

**Published:** 2024-09-26

**Authors:** Wei Liang, Xin Zhao, Pengpeng Wu, Yuxin Li, Shuai Lv

**Affiliations:** 1 College of Mechanical and Electrical Engineering, Changchun University of Science and Technology, Changchun 130022, China; 2 Zhongshan Institute, Changchun University of Science and Technology, Zhongshan 528437, China

**Keywords:** stepless parachute opening, finite element analysis, real-time measurement, wireless data transmission, static calibration

## Abstract

Spacecraft recovery technology is crucial in the field of aerospace, in which the parachute plays a key role in slowing down the descent speed of the spacecraft and realizing a smooth landing. In order to construct a dynamically adjustable parachute deployment strategy, it is necessary to measure the parachute dynamic load accurately in real-time. However, the existing sensor measurement scheme makes it difficult to meet the measurement requirements due to its large structure and complex wiring. In order to meet the current demand for real-time measurement of parachute cords dynamic load, a miniature measuring instrument is designed. According to the function and technical requirements of the miniature measuring instrument, the hardware modules of the acquisition system are selected and designed, and the integration debugging and performance optimization of the microcontroller module, A/D sampling module, signal acquisition circuit, and power supply module are carried out. The software of the parachute cords tension acquisition system based on the miniature measuring instrument is developed. The Load Cell is modeled by using SolidWorks 2022 and statically analyzed by using Ansys 2022 R1 Workbench finite element analysis software. Then the final structure of the Load Cell and the pasting position of the strain gauge are determined through the results analysis as well as experimental verification. The hardware module of the signal acquisition system for the miniature measuring instrument is then encapsulated. The force value of the miniature measuring instrument is calibrated and tested many times by using the microcomputer-controlled electronic universal testing machine. The experimental results show that the designed miniature measuring instrument has accurate data, strong stability, and good real-time performance, which meets the demand for real-time accurate measurement of miniature measuring instruments, and can provide reliable data for parachute cords parameter validation and stepless unfolding design.

## 1. Introduction

Spacecraft recovery technology plays a crucial role in the aerospace field, and the parachute system is a key component for decelerating and ensuring the smooth landing of spacecraft [1]. In this process, accurately measuring the dynamic loads on the parachute cords during deployment is essential for designing effective recovery strategies. However, current measurement technologies face limitations in terms of real-time capabilities, device size, and application flexibility, making it challenging to meet the demands of modern space missions.

Currently, technologies for measuring dynamic loads on ropes are often applied to tension measurement of yarns or steel cables, typically relying on wired sensor systems or large measurement devices [2,3,4]. While these systems perform well in certain fixed or semi-fixed scenarios, they are less portable and suitable for the dynamic load measurements required during parachute deployment, due to their large size and weight, which are not ideal for the stringent requirements of spacecraft parachute systems.

In terms of tension sensors for parachute systems, several researchers have conducted studies. For example, Cheng Yuanlu et al. designed a tension sensor for traction parachute ropes [5], while Guo Ruipeng et al. proposed a non-invasive tension sensor [6]. These sensors can be non-destructively integrated into the parachute system, but the measurement data still needs to be transmitted via wired connections, which can be inconvenient during the parachute deployment process.

Recent research has seen many scholars attempt to develop miniaturized wireless transmission devices to address these issues [7,8]. For instance, Fields proposed a wireless, non-invasive load sensor instrument kit that can be clamped onto existing suspension lines to capture load information on each line. However, this load sensor is only suitable for small parachutes and may not withstand the dynamic impact forces in extreme environments encountered during spacecraft deployment.

Given the current limitations in measurement technology, developing a small, portable device with wireless data transmission capability is particularly important. Especially in parachute systems, real-time and accurate measurement of dynamic loads on the parachute cords is crucial for optimizing parachute deployment strategies and ensuring the safe recovery of spacecraft. Traditional staged parachute deployment systems [9] usually require pre-determined deployment sequences and quantities, which limit the system’s adaptability to varying atmospheric conditions and flight states. Therefore, a continuously adjustable parachute deployment system that can adapt to real-time environmental conditions is a promising research direction. To achieve this, precise dynamic load measurement of the parachute cords is essential.

This study has designed and developed a miniaturized wireless measurement instrument based on a Load Cell body structure. This device can measure dynamic loads on the parachute lines during deployment in real-time and transmit the measurement data to a host computer via a wireless data transmission module. This design not only significantly reduces the size and weight of the device but also simplifies the system’s installation and operation through wireless transmission technology. Compared to existing technologies, the proposed device offers significant innovations in the following aspects:

1. Miniaturized Design: Utilizing integrated circuits and optimized Load Cell body structure design, the device can be embedded into the parachute line system without interfering with the parachute deployment process.
2. Wireless Transmission: Eliminating the constraints of traditional wired data transmission, real-time data transmission through wireless modules greatly enhances the system’s flexibility and portability.
3. Real-Time Measurement and Feedback: The device can transmit data in real-time to the host computer for processing and display while the parachute cords endure dynamic loads, providing crucial data support for subsequent parachute design and optimization.

With these innovations, this miniaturized measurement instrument provides important technical support and data sources for future continuous parachute deployment design and other dynamic load measurements.

## 2. Overall Structure of Miniature Measuring Instrument

### 2.1. Overview of Miniature Measuring Instrument

**Figure 1:**
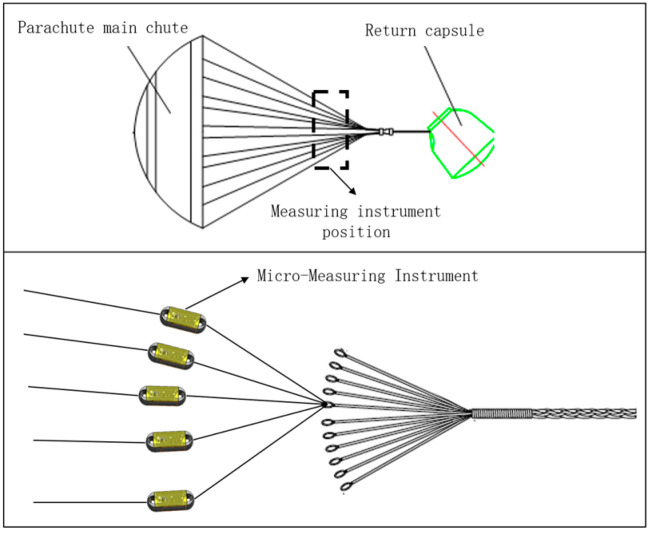
Schematic of Miniature Measuring Instrument Installed in a Parachute.

The instrument is installed on the parachute cords in the form of “tandem”, and through the signal acquisition system, the strain signal of the Load Cell is converted into an electrical signal, and the collected data are sent wirelessly to the upper computer test software, which processes and saves the data. The schematic diagram of the miniature measuring instrument installed in the parachute is shown in Figure 1.

**Figure 2:**
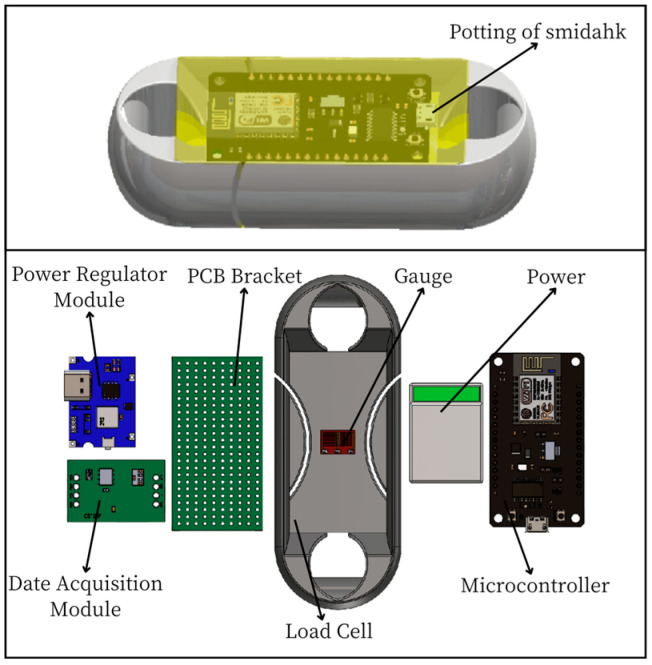
Schematic of Miniature Measuring Instrument.

The miniature measuring instrument includes a Load Cell and signal acquisition system, through the strain gages pasted on the Load Cell to form a Wheatstone bridge [10,11] to transform the strain signal generated by the Load Cell into an electrical signal, the data acquisition module will save the data to the micro-controller module, which will send the data through the wireless transmission module to the upper computer test software. Figure 2 is the schematic of the miniature measuring instrument.

### 2.2. Overall Structure of Miniature Measuring Instrument Design Requirements

To fulfill the need for real-time measurement of parachute cords loads, the miniature measuring instrument has to be integrated into the parachute cords in a non-destructive way that does not affect its performance. The signal acquisition system must ensure real-time, accurate and stable data collection, transmission, and storage for future analysis. The miniature measuring instrument shall incorporate the following features:

1. The miniature measuring instrument meets the needs of miniaturization and lightweight;
2. The miniature measuring instrument is capable of real-time collection of parachute cord load data;
3. The miniature measuring instrument can be calibrated by software;
4. The miniature measuring instrument can transmit the data wirelessly to the upper computer test software.

A certain model parachute has a load capacity of 6.5 kN per suspension line. According to the sensor design method, the sensor’s range needs to be 150% of the full scale (FS). Therefore, the maximum range of the sensor is 9.75 kN. Based on the precision requirements for the miniature measuring device, the load capacity of the miniature measuring device developed in this paper is defined as 8000 N.

The miniature measuring instrument not only needs to meet the above functional requirements but also needs to meet the expected indicators which are shown in Table 1.

### 2.3. R&D Process

**Figure 3:**
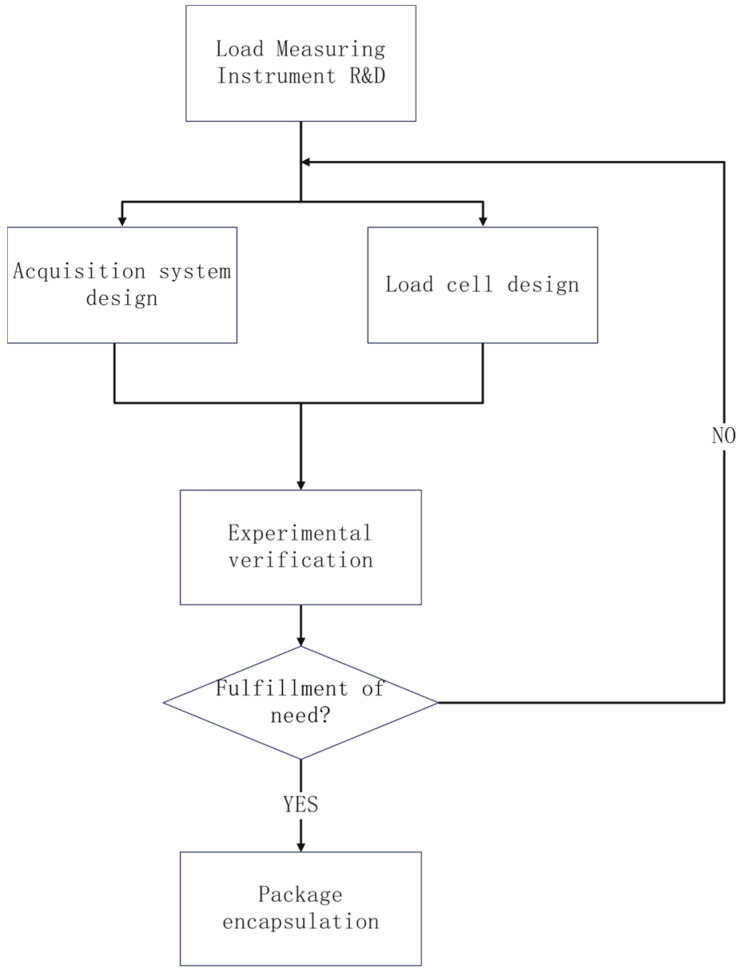
Parachute Cords Dynamic Load Measuring Instrument R&D Program.

In order to meet the required functions and main technical indicators, the design and development of the miniature-measuring instrument is divided into two major parts: signal acquisition system design and Load Cell design. These two parts interact with each other and an appropriate design research program is proposed to meet the final design requirements, as shown in Figure 3.

### 2.4. Analysis of Expected Problems (Expected Challenges/Potential Problems)

Comprehensively analyzing the design and functional requirements of the parachute cords load measuring instrument, the researchers need to focus on solving the following four key technical problems:

1. Microspace data acquisition: integrating the microcontroller module, data acquisition module, strain gauge, power supply module, and wireless transmission module in a limited space to ensure measurement accuracy, sampling frequency, and data transmission requirements.
2. Load Cell design: the Load Cell needs to withstand high impact loads while maintaining strain gauge accuracy and ensuring the stability of the electronic component area to guarantee the stability of the circuit package.
3. Encapsulation of electronic instruments in high-load environments [12]: the technology to ensure that the electronic components can still operate normally and stably under impact forces.
4. Testing and calibration methods: developing testing and calibrating methods for the miniature measuring instrument to ensure data accuracy and evaluate its performance through actual testing.

## 3. Design of the Load Cell

### 3.1. Load Cell Design Requirements

The measuring principle of the miniature measuring instrument is that when the Load Cell is deformed under the action of the pulling force generated by the parachute cords, a tensile and compressive strain in the central web is formed [13]. By pasting a resistance strain gauge in a larger strain area, the conversion and acquisition of the deformation signal to the electrical signal can be carried out. Thus, the design requirements should be as follows:

1. High precision, good stability, durability, and other advantages;
2. Integrating into a parachute structure in series without influencing the normal opening of the parachute;
3. Miniaturization and lightweight;
4. The Load Cell is capable of accommodating appropriate deformations when subjected to loads.

### 3.2. Design of Load Cell I

**Figure 4:**
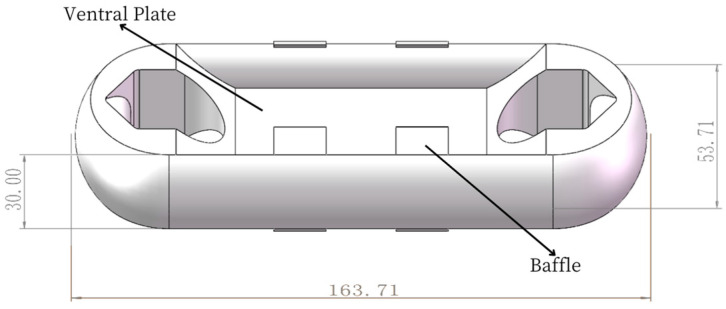
Design of Load Cell Scheme I.

The Load Cell I is designed to be an integrated structure and the two end earrings are connected with the parachute cords. The middle concave web plate is used for installing a strain gauge and encapsulating electronic components of an acquisition system, and the eight protruding “baffles” are used to protect electronic components [14]. SolidWorks 2022 software is used for 3D modeling, and the design size of Load Cell Scheme I is 163. 71 mm × 53.71 mm × 30 mm, as shown in Figure 4.

It is constructed from AI304 stainless steel, chosen for its excellent corrosion resistance, high strength, and durability. Additionally, AI304 stainless steel’s good machinability facilitates the complex design and precision required for this Load Cell, ensuring reliable performance and long-lasting structural integrity. The physical and mechanical properties of the material are as follows in Table 2.

#### 3.2.1. Linear Static Analysis of Load Cell Structure I

The measurement principle of the miniature measuring instrument is that the Load Cell undergoes corresponding Load Cell deformation with the applied force, without producing plastic deformation. Therefore, the Load Cell of the miniature measuring instrument requires a linear static analysis, where the material, structure, and state of the object must meet linear conditions. Under these conditions, the relationship between model load and deformation will exhibit a linear variation.

**Figure 5:**
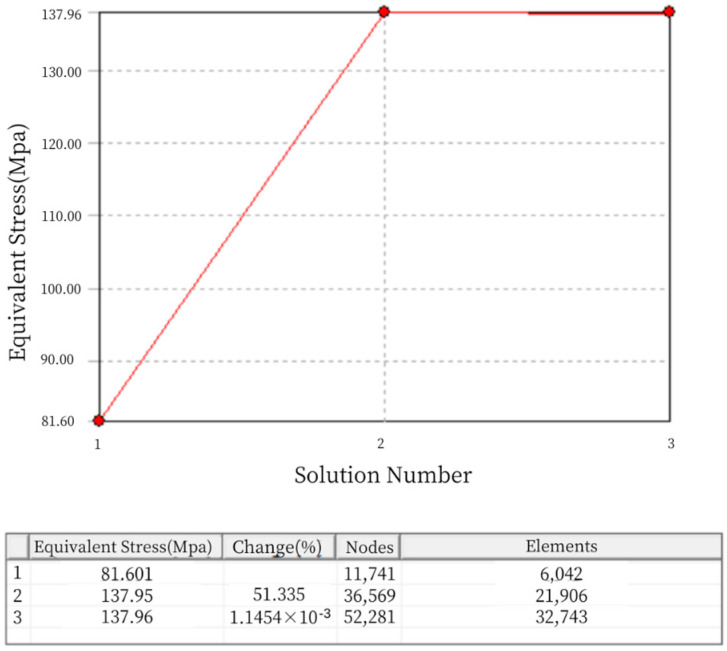
Refinement Convergence Results of Adaptive Mesh Division for Load Cell Structure I.

When establishing the finite element model, this paper adopts the adaptive meshing technology provided by Workbench. The adaptive meshing relies on Ansys 2022 R1 to estimate the error caused by the mesh division, which determines whether the mesh division is adequate. If the mesh is not fine enough, the program will automatically refine the mesh to reduce the error. During the refinement of the adaptive mesh, the distribution of mesh points is coupled with the physical behavior to improve the accuracy and precision of the simulation results. The process of adaptive meshing in Workbench is as follows:

1. Enter the pre-processing phase of static analysis, complete the definition of materials, import the geometric model, and set the boundary conditions.
2. Perform an initial mesh division using the default method, defining the overall mesh size control, with the maximum size not exceeding 5 mm.
3. Set the solution properties, with the default subdivision depth set to 2, and a maximum of 5 mesh subdivisions, meaning the mesh will be divided up to 5 times.
4. In post-processing, insert the convergence object for the region of interest and set the convergence error criteria, then start the calculation. During the iterative process of adaptive meshing, the mesh element size will automatically adjust (within the size range specified in step 2) until the maximum number of iterations (set in step 3) or the error satisfies the specified value (the allowed maximum error set in step 4). Obviously, when setting the convergence error criteria, the smaller the value, the more precise the simulation results. However, considering the simulation computation load and solving time, the convergence error is usually set to no more than half of the default value, which is 20%. For example, in the equivalent stress solution domain, a convergence object is inserted, and the maximum allowable error is set to 5%. After the calculation is completed, the refined convergence results can be viewed, as shown in Figure 5.

**Figure 6:**
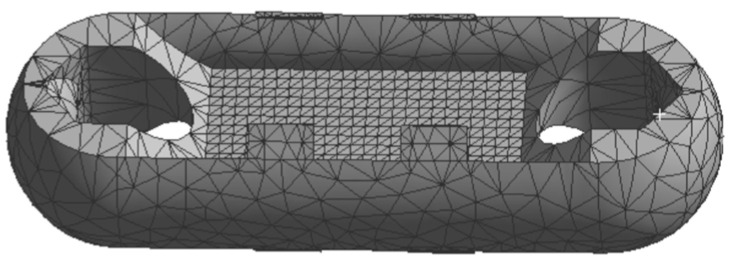
Mesh Division Diagram of Load Cell Structure I.

It can be seen that the mesh division in the entire static analysis underwent two refinements, reaching a convergent state. In Figure 5, the “change (%)” column represents the current convergence error. After two mesh refinements, the error becomes negligible, meeting the requirement of less than 5% error specified during the setup. Therefore, the iteration of the adaptive mesh division concludes. The final number of mesh nodes is 52,281, and the number of elements is 32,743, as shown in Figure 6.

**Figure 7:**
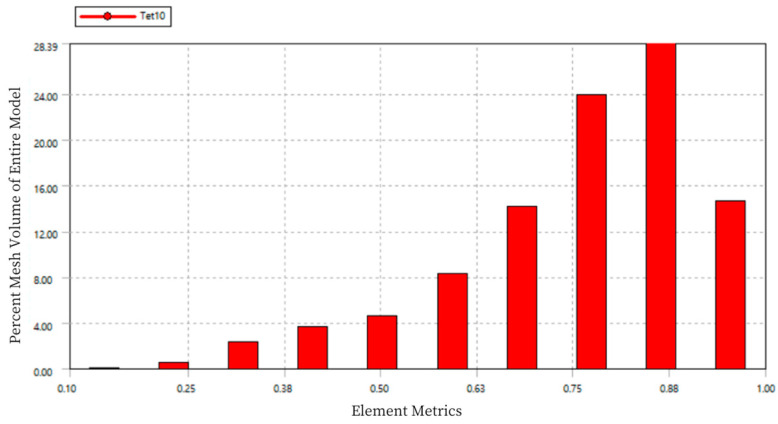
Mesh Quality Statistics Diagram of Load Cell I.

Using Workbench, on one hand, adaptive mesh refinement can be carried out based on specified standards, avoiding the repetitive task of manually redefining the mesh after each result. On the other hand, mesh quality checks can be performed to ensure the quality of the mesh and the accuracy of the simulation, as shown in Figure 7.

In the chart, the horizontal axis “Element Metrics” represents the comprehensive quality measurement standard, and the vertical axis “Percent Mesh Volume of Entire Model” represents the percentage of the total model volume for each mesh quality. The quality of the mesh elements is measured on a scale from 0 to 1, where a value of 0 indicates a cell with zero or negative volume, which should be avoided in the mesh division. A value of 1 represents a perfect cube or square, which is the ideal mesh. As seen in the figure, the mesh quality is concentrated between 0.75 and 0.9, indicating good mesh division quality.

#### 3.2.2. Boundary Condition Setup and Static Simulation Results Analysis

**Figure 8:**
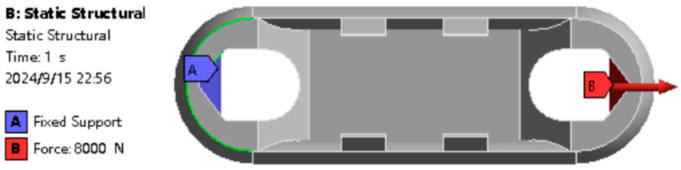
Boundary Condition Setup.

One side of the earring is fixed, and an 8000 N force is applied to the other side of the earring. The finite element analysis results of the Load Cell Scheme I are shown in Figure 8.

**Figure 9:**
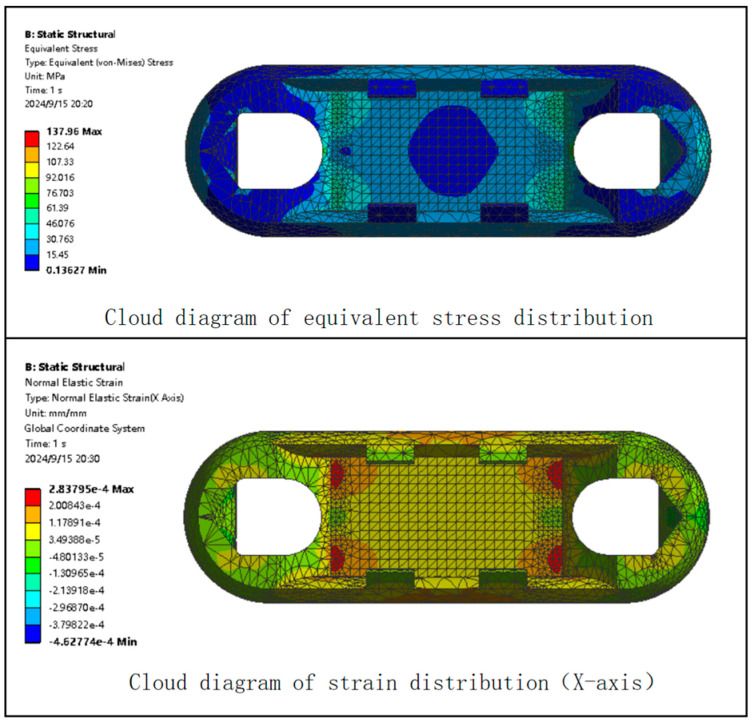
Strain Diagram of Load Cell Scheme I.

As shown in Figure 9, after the Load Cell Scheme I bears a force of 8000 N, its maximum stress is 137.96 MPa, which is below the material’s maximum yield strength of 206.807 MPa, indicating that the material meets the structural requirements. Therefore, the range of the designed miniature measuring instrument can theoretically reach 8000 N.

As shown in Figure 9, after bearing a force of 8000 N, the maximum strain in Load Cell Scheme I is 2.84 × 10^−4^ mm/mm. The maximum strain occurs at the four corners of the Load Cell’s web position. Thus, the location with the maximum strain in the Load Cell can be selected as the position for attaching the strain gauges.

The prototype of the miniature measure instrument is obtained by assembling and welding the hardware module I of the signal acquisition system (see Section 4.2) and Load Cell I. The experimental verification test is conducted on the universal testing machine (see Section 5.1). The experimental results show that the volume size of Load Cell I is so large that it is difficult to paste the strain gauge in the strain area. Thus, a structural optimization design of the Load Cell I is necessary.

### 3.3. Optimized Design of Load Cell

#### 3.3.1. Design of Load Cell

**Figure 10:**
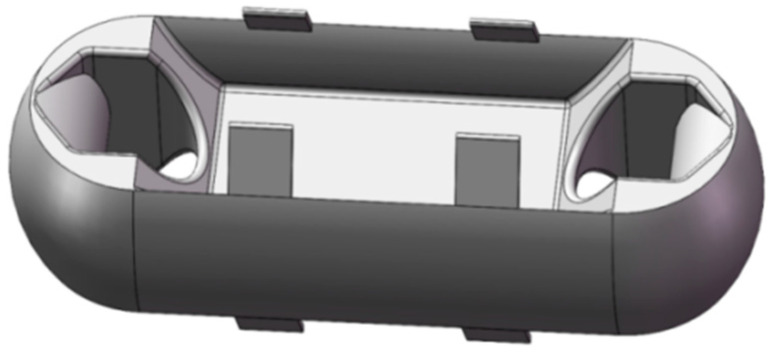
Load Cell Scheme II.

**Figure 11:**
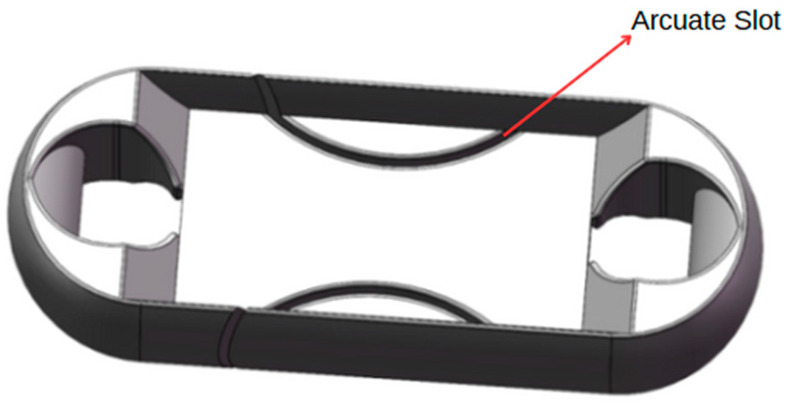
Load Cell Scheme III.

The design of Load Cell of the miniature measuring instrument directly determines the performance of the instrument. In this paper, the structural optimization design is carried out according to the Load Cell I. The hardware module of that acquisition system is packaged on both sides of the Load Cell by adopting two assembly schemes. The optimized Load Cells are called Load Cell Scheme II and Load Cell Scheme III. In Load Cell Scheme II, the battery is on one side, the microcontroller, the A/D sampling module, and the charging module are on the other side. The Load Cell Scheme III adopts one side of the microcontroller and the battery, and the other side of the A/D sampling module and the charging module. Load Cells II and III are shown in Figure 10 and Figure 11.

On the basis of the Load Cell Scheme I, the Load Cell Scheme II carries out the reduction in the web thickness, the reduction in the overall size, and the angle changes of the baffle plate to complete the optimization of the Load Cell. On the basis of the Load Cell Scheme I, the Load Cell Scheme III is optimized by slotting, reducing the overall size, increasing the thickness of the web, and removing the baffle plate. Finally, the size of Load Cell II is 119. 47 mm × 43.47 mm × 30 mm, and the size of Load Cell III is 96 mm × 36 mm × 22 mm.

#### 3.3.2. Statics Analysis of Load Cell II

After determining the Load Cell form, static analysis of the two optimized Load Cell schemes is performed using the finite element analysis software Ansys 2022 R1 Workbench to identify the region of maximum deformation in the Load Cell.

Based on the finite element analysis steps used for Load Cell I, static analysis is conducted for Load Cell II.

**Figure 12:**
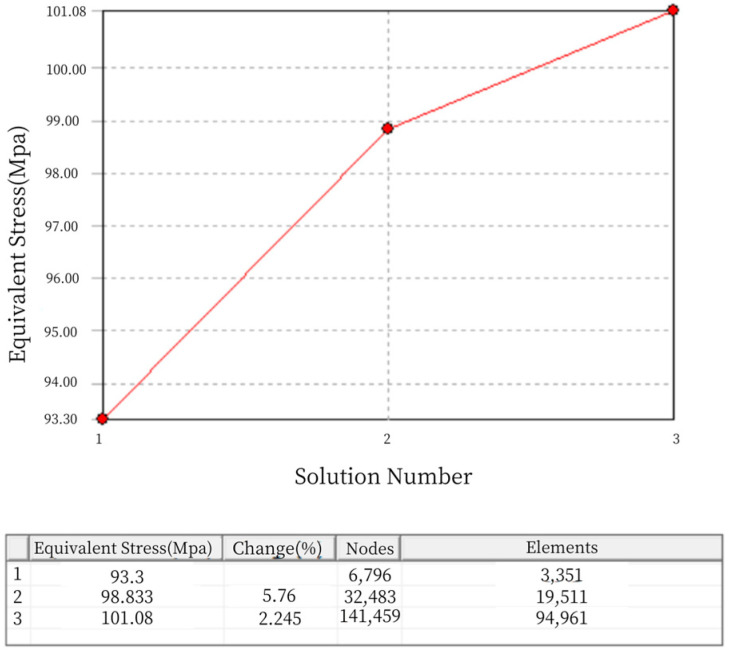
Refinement Convergence Results of Adaptive Mesh Division for Load Cell II.

**Figure 13:**
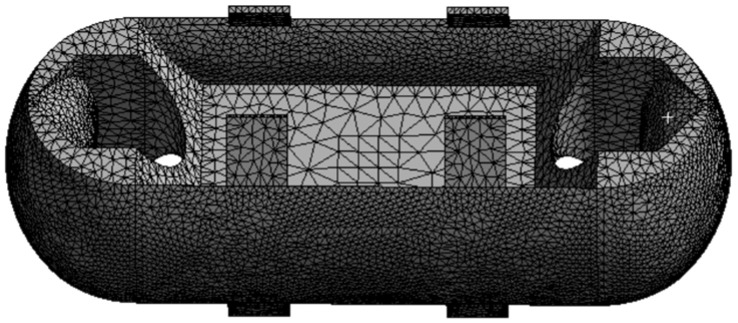
Mesh Division Diagram of Load Cell II.

It can be seen that the mesh division underwent two refinements during the static analysis, reaching a convergent state. In Figure 12, the “change (%)” column represents the current convergence error. After two mesh refinements, the error is 2.245%, which meets the specified requirement of less than 5% error. Therefore, the iteration of the adaptive mesh division concludes. The final number of mesh nodes is 141,459, and the number of elements is 94,961, as shown in Figure 13.

**Figure 14:**
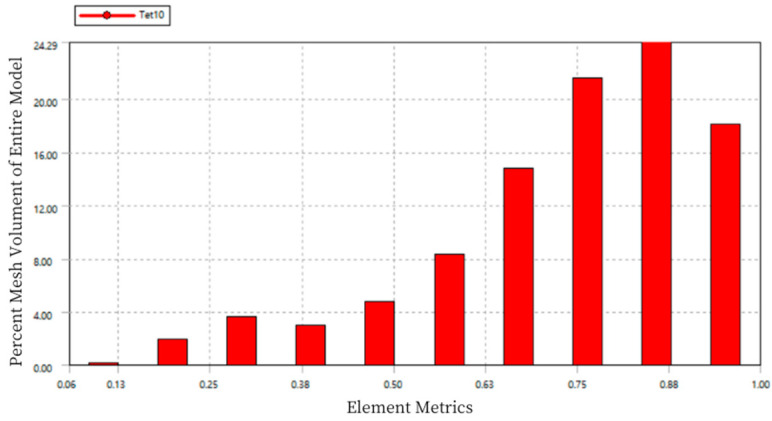
Mesh Quality Statistics Diagram of Load Cell III.

The quality of the mesh elements is measured on a scale from 0 to 1, where a value of 0 represents elements with zero or negative volume, which should be avoided in the mesh division. A value of 1 represents a perfect cube or square, which is the ideal mesh. As shown in Figure 14, the mesh quality is concentrated between 0.75 and 0.9, indicating good mesh division quality.

**Figure 15:**
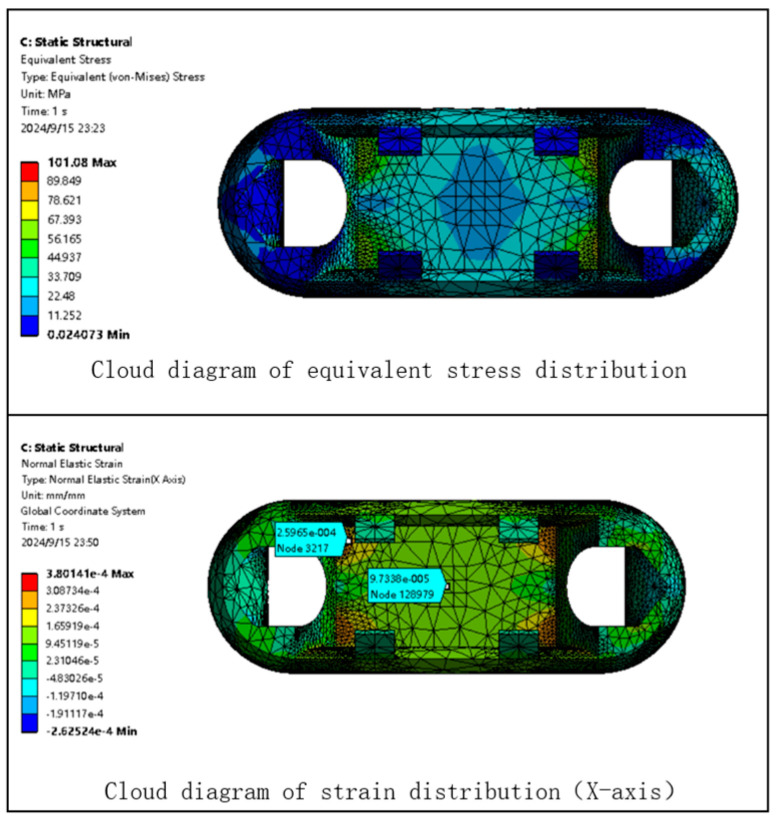
Static Analysis Cloud Diagram of Load Cell II.

Based on the static analysis of Load Cell I, a static analysis is performed on Load Cell II under the same conditions. The finite element analysis cloud diagram for Load Cell II is shown in Figure 15.

As shown in Figure 15, the maximum stress experienced by Load Cell II is 101.08 MPa, which is less than the yield strength of the selected material. Theoretically, this material can meet the measurement range of the miniature measuring instrument. The stress concentration area in Load Cell II is located at the four corners of the web of the Load Cell, where the strain is 260 με. The strain in the center area of the web is 97 με.

#### 3.3.3. Static Analysis of Load Cell III

Based on the finite element analysis steps of Load Cell I, a static analysis is carried out for Load Cell III.

**Figure 16:**
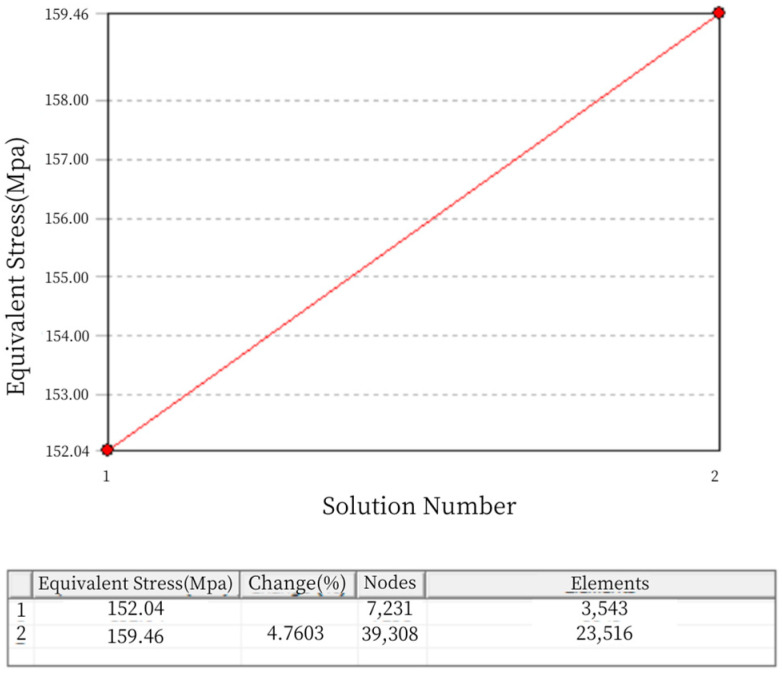
Adaptive Mesh Refinement Convergence Results for Load Cell III.

**Figure 17:**
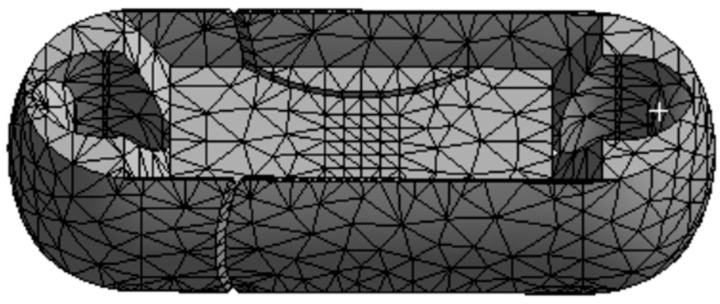
Mesh Generation Diagram for Load Cell II.

As shown, the mesh refinement process for the static analysis of Load Cell III was carried out twice and reached a convergent state. The “change (%)” column in Figure 16 indicates the current convergence error. After one mesh refinement, the error was 4.7603%, meeting the specified requirement of an error of less than 5%. Thus, the iterative process for adaptive mesh refinement has ended. The final mesh division has 39,308 nodes and 23,516 elements, as shown in Figure 17.

**Figure 18:**
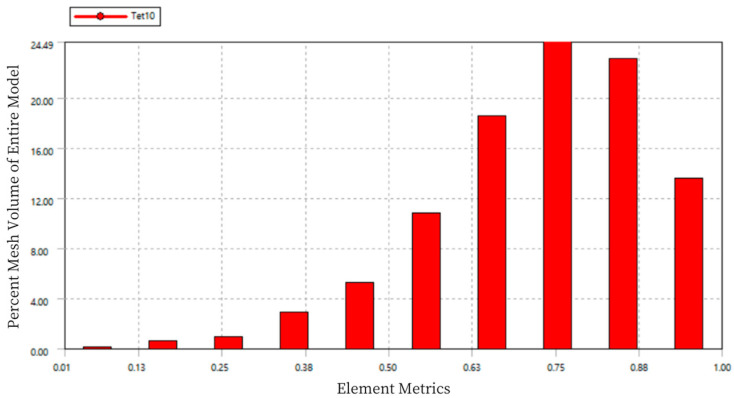
Mesh Quality Statistics for Load Cell I.

The mesh quality statistics are measured on a scale of 0 to 1, where a value of 0 indicates that the element volume is zero or negative (which should be avoided), and a value of 1 represents a perfect cube or square, which is ideal. As shown in Figure 18, the mesh quality is concentrated between 0.7 and 0.9, indicating a good quality of mesh division.

**Figure 19:**
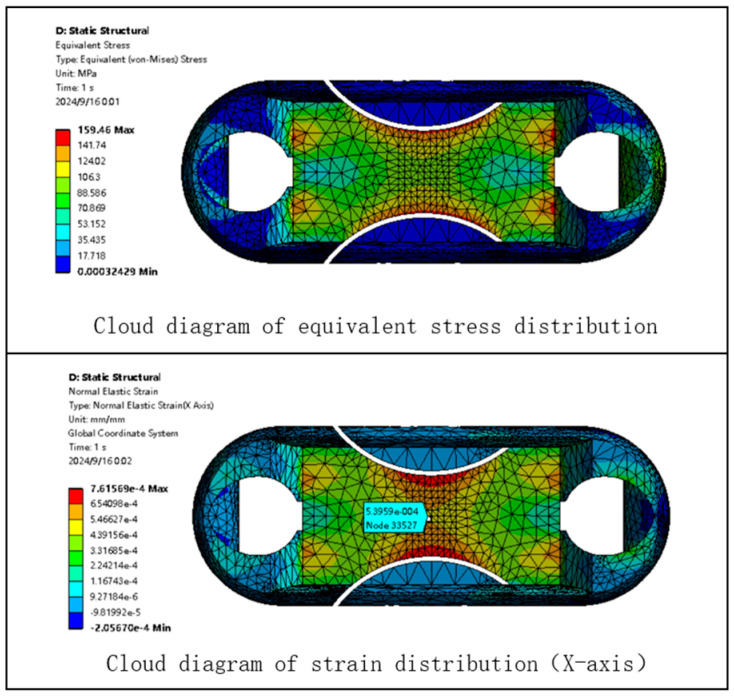
Static Analysis Contour Plot for Load Cell III.

Based on the static analysis of Load Cell I, a similar static analysis is conducted for Load Cell III under the same conditions. The finite element analysis contour plot for Load Cell III is shown in Figure 19.

As shown in Figure 19, the maximum stress experienced by the Load Cell body structure III is 159.46 MPa, which is below the yield strength of the selected material. This indicates that the material is theoretically capable of meeting the measurement range requirements for the miniature measuring instrument. The stress concentration area for Load Cell III is located at the center of the web plate of the Load Cell body structure, where the strain is 540 με.

#### 3.3.4. Strain Gage Selection and Pasting Position Determination

**Figure 20:**
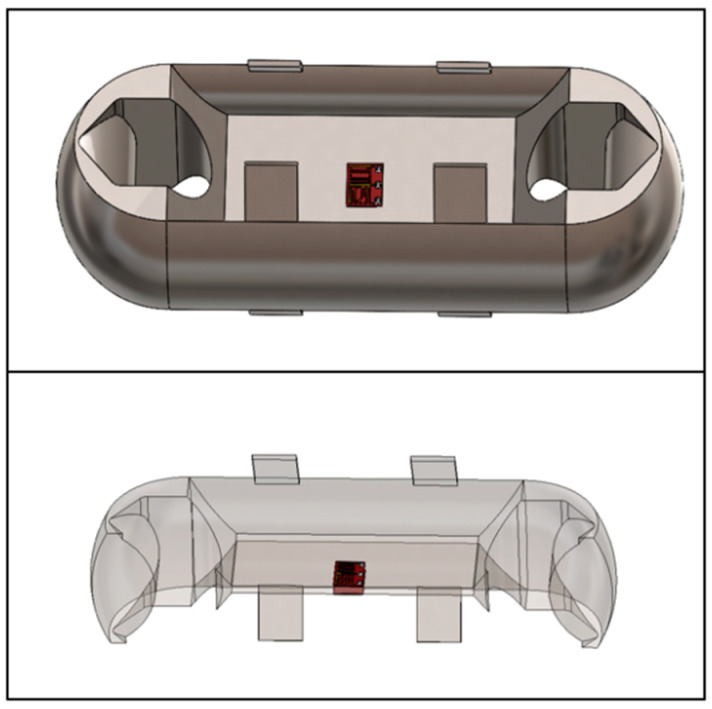
Strain Gage Pasting Position in Load Cell Scheme II.

**Figure 21:**
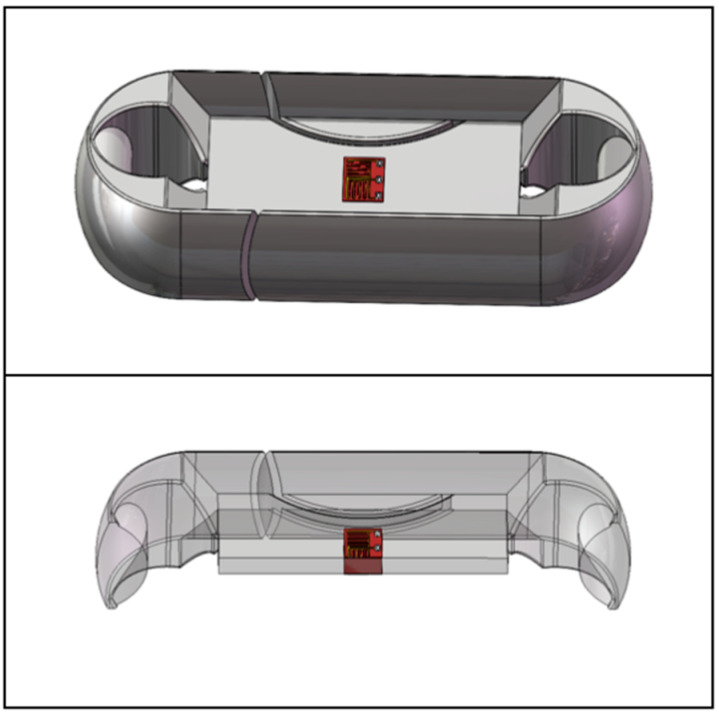
Strain Gage Pasting Position in Load Cell Scheme III.

In this paper, the “T” type strain gauge of model BF-350-3BB-A is used. In order to eliminate the influence of the temperature error and further improve the sensitivity of the miniature measuring instrument, two half-bridge strain gages are selected to form a full bridge [15]. According to the principle of “stress concentration”, it is difficult to paste the strain gage on the Load Cell II. Therefore, the strain gauge is pasted at the center of the web with uniform stress. The pasting position of the strain gauge of the Load Cell is shown in Figure 20 and Figure 21.

By using the “probe” function in Ansys 2022 R1, the strain is collected in the area where the strain gauge is attached. Collect every 600 N for 14 times, and collect 20 points each time. Take the average value of the collected data and draw the line chart.

**Figure 22:**
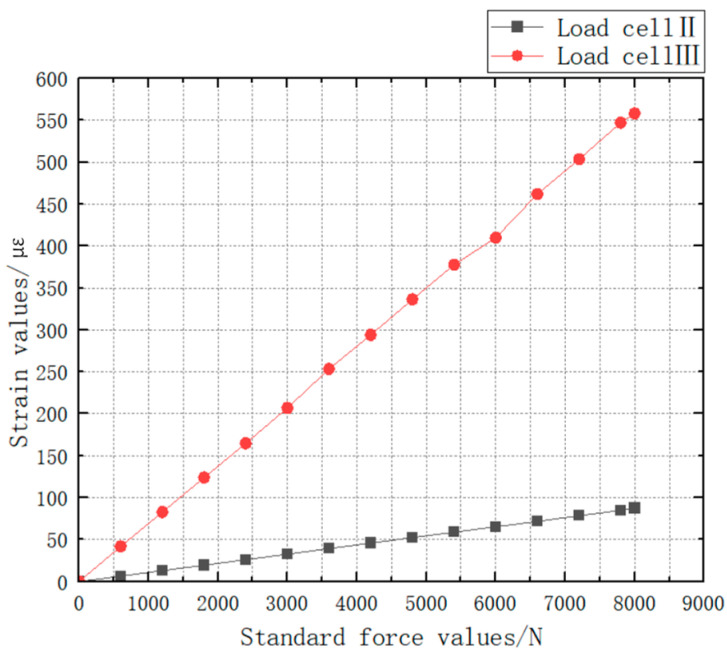
Axial Strain at the Center of Load Cell Scheme II and Scheme III.

From Figure 22, it can be seen that the strain value of Load Cell II is much smaller than that of Load Cell III under the same force loading. In order to determine the final Load Cell scheme, experimental verification is required (see Section 5.2 for experimental verification of Load Cell scheme).

### 3.4. Theoretical Calculation of Strain Values for Load Cell III

The axial and transverse strain values of Load Cell III are summarized in Table 3. A positive value indicates tensile strain measured by the strain gauge, while a negative value indicates compressive strain.

**Figure 23:**
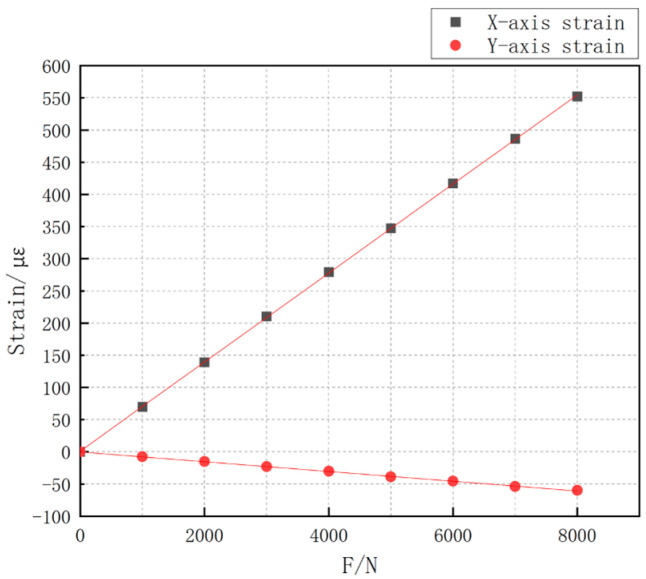
X-axis and Y-axis strains at the center of the Load Cell structure III.

Figure 23 visually shows that the relationship between the tensile force value and the strain value exhibits a linear trend, indicating that the linearity of the sensor is theoretically good.

Based on the fitted straight line, the relationship between the load applied to the strain gauge and the corresponding strain is as follows:

(1) $$\varepsilon_{x}=6.9\times 10^{-8}F+1.12\times 10^{-6}$$

(2) $$\varepsilon_{y}=-7.58\times 10^{-9}F-1.54\times 10^{-7}$$

According to the principle of the Wheatstone Bridge, the following can be obtained:

(3) $$V_{out}=\frac{1}{4}U(∆R_{1}-∆R_{2}-∆R_{3}+∆R_{4})/R$$

where:

(4) $$\left\{\begin{array}{c} \frac{{∆R}_{2}}{R}=\frac{{∆R}_{3}}{R}=K\varepsilon_{x}\\ \frac{{∆R}_{1}}{R}=\frac{{∆R}_{4}}{R}=K\varepsilon_{y} \end{array}\right.$$

That is:

(5) $$V_{out}=\frac{1}{2}UK(\varepsilon_{x}-\varepsilon_{y})$$

Substituting Equations (1) and (2) into (5), with an input voltage of 3300 mV and assuming a strain gauge sensitivity coefficient of 2, the relationship between the miniature measuring instrument and the tensile force is obtained as:

(6) $$V_{out}=2.53\times 10^{-4}F-4.21\times 10^{-3}$$

## 4. Microspace Data Acquisition

### 4.1. Signal Acquisition System Design Requirements

The function of the signal acquisition system mainly includes the real-time acquisition of parachute cord tension and the data transmission to the upper computer through wireless communication. The microcontroller module, signal acquisition module, strain gauge, power supply module, and wireless transmission module must be integrated into the Load Cell within a limited space, and at the same time, the requirements of measurement accuracy, sampling frequency, and data transmission should be met. So the signal acquisition system needs to meet the following functions:

1. The hardware structure of the signal acquisition system needs to be compact;
2. The signal acquisition system needs to be both high performance and low power consumption;
3. The miniature measuring instrument is battery-powered and supports direct charging without removing the battery. The endurance time is about 20 min;
4. The sampling rate of the miniature measuring instrument is 1 kHz, which can completely record the change of the pull force of the parachute cords in the process of opening and straightening, and conduct wireless data transmission.

### 4.2. Signal Acquisition System Design and Performance Test

**Figure 24:**
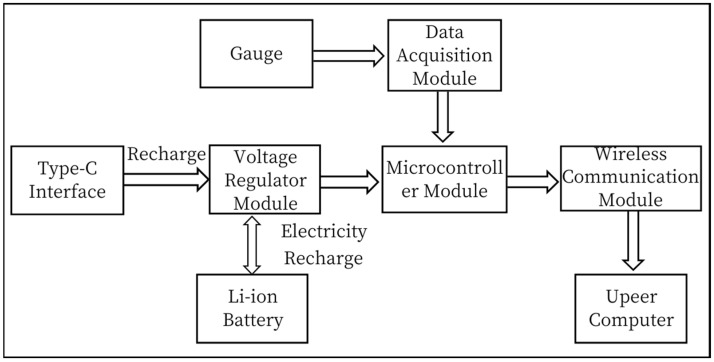
Schematic Diagram of Signal Acquisition System.

The signal acquisition system of the miniature measuring instrument mainly completes the functions of data acquisition and wireless data transmission of the dynamic load of parachute cords. It mainly includes a microcontroller module, A/D acquisition module, strain gauge, power supply module, and wireless communication module. Figure 24 is a schematic diagram of the signal acquisition system.

#### 4.2.1. Signal Acquisition System Hardware Module Selection and Design

In order to meet the demand for the miniature measuring instrument, the design scheme of module separation is adopted. The hardware modules are arranged on both sides of the Load Cell and connected by connecting lines to solve the problem that the overall volume of the circuit board is too large to be installed on the Load Cell.

**Figure 25:**
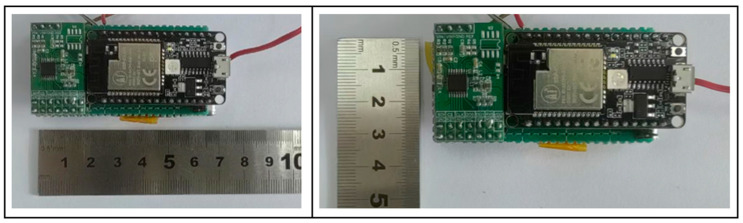
Photos of Signal Acquisition System Hardware Module I.

According to the design requirements, the results of the selection and design of the signal acquisition system hardware are as follows:

According to Table 4, the relevant electronic components are assembled and welded, and the photos of the acquisition system hardware module I with the relevant dimensions 67.8 mm × 33.84 mm × 21.63 mm (L × W × H) are shown in Figure 25.

The performance parameters of the signal acquisition system hardware module I are shown in Table 5 below.

#### 4.2.2. Upper Computer Test Software Design and Development

**Figure 26:**
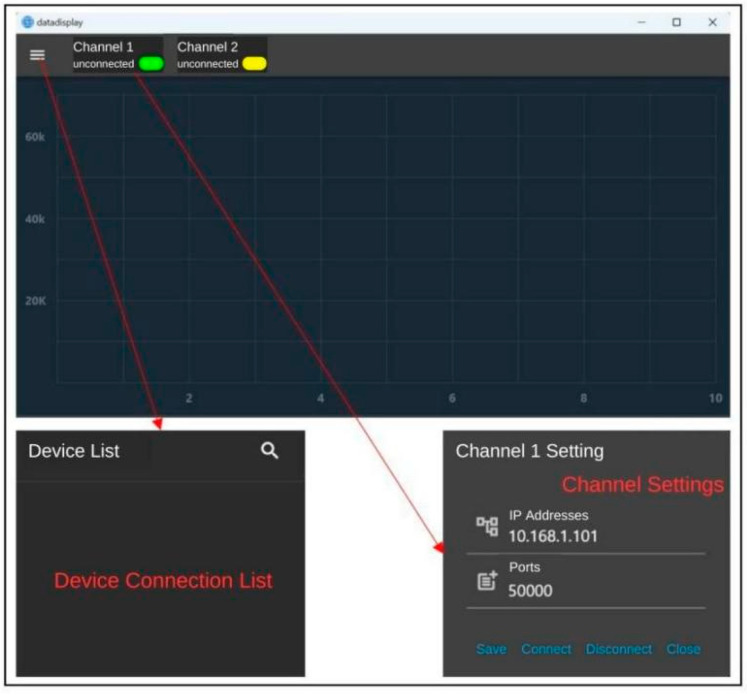
Signal Acquisition System Software Interface.

In this paper, Visual Studio 2022 software is used to write the upper computer software [16,17] with the test software interface shown in Figure 26. Click on the “instrument list” to select the instrument to be connected, and click on “channel 1” to set up the instrument channel. After the instrument is successfully connected, the upper computer test software will plot curves according to the data collected and update them in real-time.

#### 4.2.3. Communication Test of Signal Acquisition System and Upper Computer Test Software

**Figure 27:**
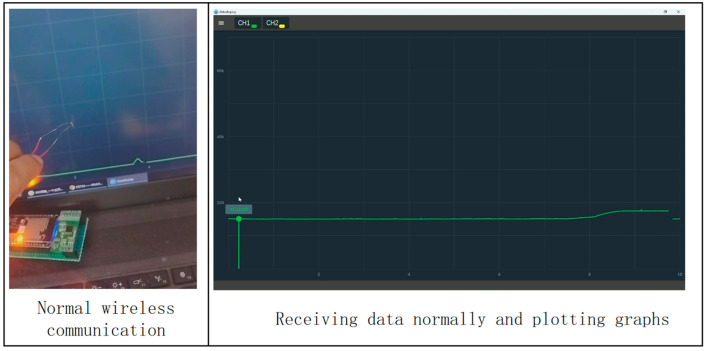
Upper Computer Software Displaying Waveforms.

Before the communication test, it is necessary to set the IP address of the hardware module of the signal acquisition system to 10.168.1.101, and at the same time, set the IP address of the wireless router, WiFi name and password, connect the computer equipped with the upper computer test software to WiFi and turn on the software, search for the list of equipment and bind it to channel 1, and receive the data in the upper computer test software after successful connection by pressing the strain gage slightly. The communication test proves that the hardware module I of the signal acquisition system and the upper computer test software can communicate normally and receive data and draw curves in real-time, as shown in Figure 27.

After the above communication test, it is known that the signal acquisition system hardware module and the upper computer test software have the following design defects and cannot meet the design requirements:

1. The size of the hardware module I of the acquisition system is too large, failing to achieve the design goal of lightweight and miniaturization.
2. The large size of the electronic components selected for the hardware selection and design of the signal acquisition system will have an impact on the design size of the Load Cell I, resulting in a very large overall size of the miniature measuring instrument.
3. The function of the upper computer test software is not perfect, unable to calibrate and save data, and the number of channels is limited to two. The range of the display interface is large, and it is not possible to visualize the experimental results.

Based on the above design defects, it is necessary to optimize the design of the signal acquisition system hardware module and the upper computer test software to meet the design requirements of the miniature measuring instrument.

### 4.3. Signal Acquisition System Optimization Design

#### 4.3.1. Signal Acquisition System Hardware Module Optimization

**Figure 28:**
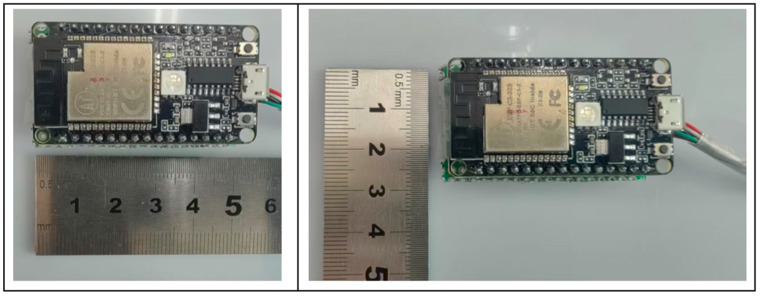
Photos of Signal Acquisition System Hardware Module II.

After the experimental test and result analysis, it is known that it is necessary to optimize the design of the hardware module of the signal acquisition system and the test software of the upper computer. The selection design of the optimized signal acquisition system hardware module (later called signal acquisition system hardware module II) is shown in Table 6. The signal acquisition system hardware module II is shown in Figure 28.

A comparison of the performance of the hardware modules of the signal acquisition system before and after optimization is shown in Table 7:

From the above comparison, it can be seen that the signal acquisition system hardware module II improves the resolution of the A/D sampling module while decreasing the sampling rate, but it can still meet the design requirements of the miniature measuring instrument.

**Figure 29:**
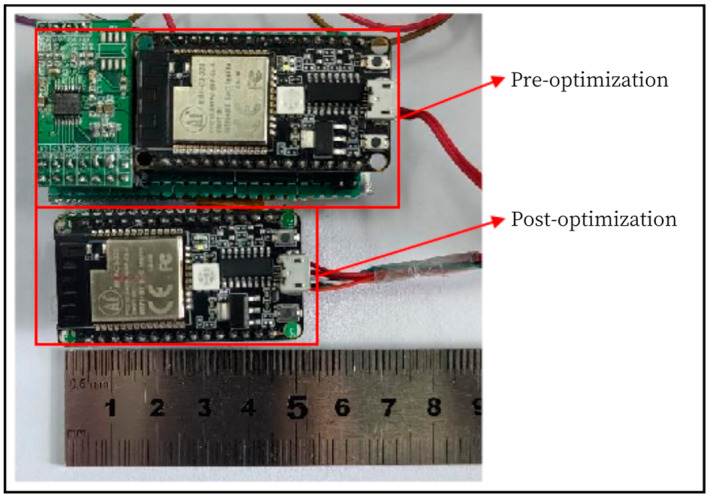
Comparison of the Signal Acquisition System Hardware Before and After Optimization.

By comparing the physical objects of the signal acquisition system modules in Figure 29, it can be seen that the volume and size of the signal acquisition system hardware module II are significantly reduced, which is in line with the design requirements of lightweight and miniaturization.

#### 4.3.2. Optimization of the Upper Computer Test Software

Aiming at the defects of the upper computer test software, the interface of the upper computer test software is optimized and the software functions are added. The optimized upper computer test software (later called parachute cords tension signal acquisition system) realizes the functions of data calibration and data saving, and at the same time, it also increases the data transmission channels to 10 and realizes real-time display of data, which makes the display of dynamic load of parachute cords more intuitive.

**Figure 30:**
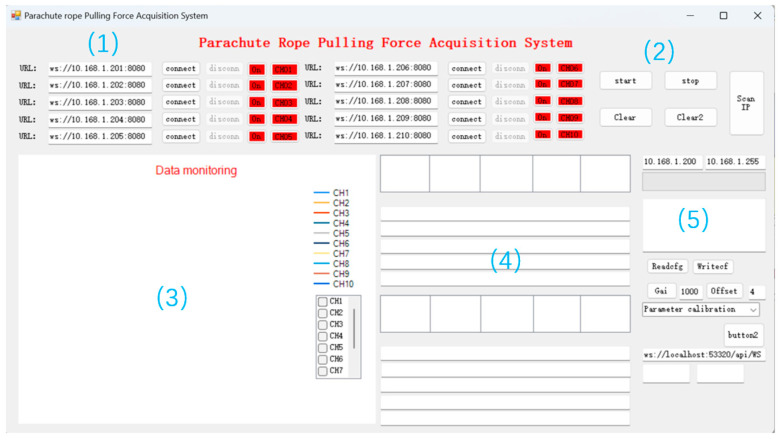
Upper Computer Software Main Interface Area Division. (1) Wireless Address Connection Area; (2) Software Operation Area; (3) Data Acquisition Plotting Display Area; (4) Real-time Data Display Area; (5) Parameter Calibration Area.

The main interface of the parachute cords tension signal acquisition system is shown in Figure 30 including five areas: Wireless address connection area, Software operation area, Data acquisition plotting display area, Real-time data display area, and Parameter calibration area.

#### 4.3.3. Communication Test of Optimized Signal Acquisition System

**Figure 31:**
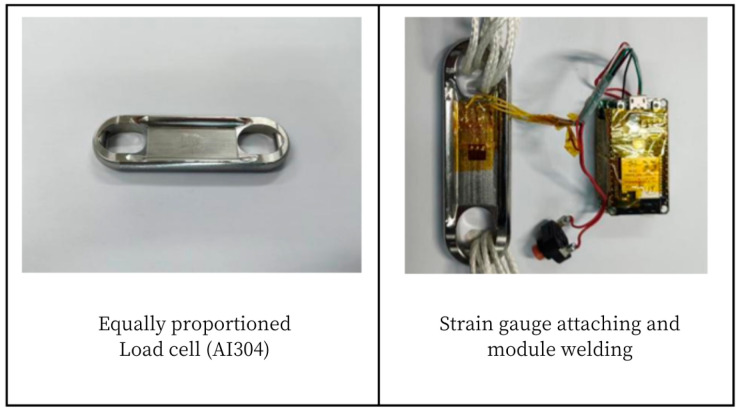
Miniature Measuring Instrument Fixed to the Load Cell Structure.

In order to make the communication test more intuitive, a scaled-down Load Cell is designed and machined with AI304 as the material. Due to the small size of the structure, the strain gauge is pasted at the center of the web, the cross-positioning line is determined at the center of the structural web with a pencil, and the signal acquisition system module is fixed on the Load Cell with insulating tape, as shown in Figure 31.

**Figure 32:**
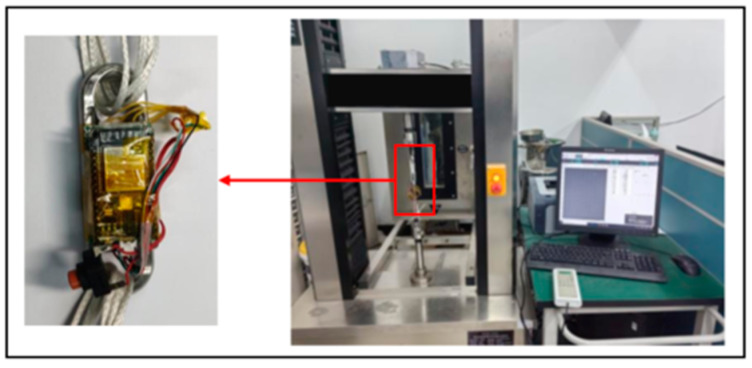
Signal Acquisition System Module II and Wireless Communication Test of Cords Tension Acquisition System.

A microcomputer-controlled electronic universal testing machine is used to carry out the experimental test on the communication of the signal acquisition system which includes the wireless communication and wireless data transmission test of the signal acquisition system module II and the parachute cords tension acquisition system software, and the function verification of the parachute cords tension acquisition system software. The wireless communication test of the signal collection system module II and the parachute cords tension acquisition system is shown in Figure 32.

**Figure 33:**
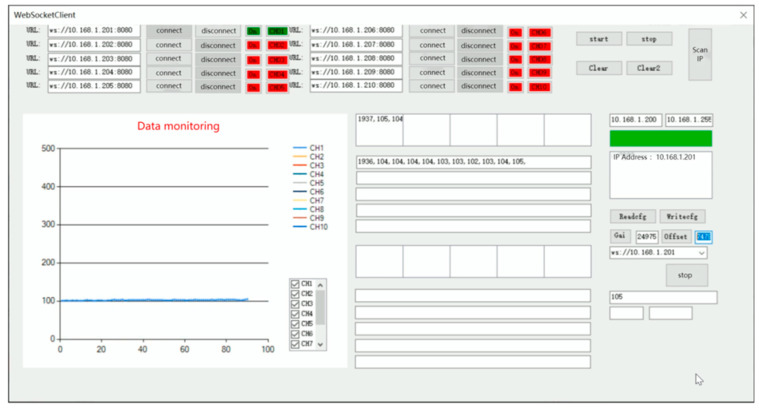
Normal Working Condition of Parachute Cords Tension Acquisition System.

**Figure 34:**
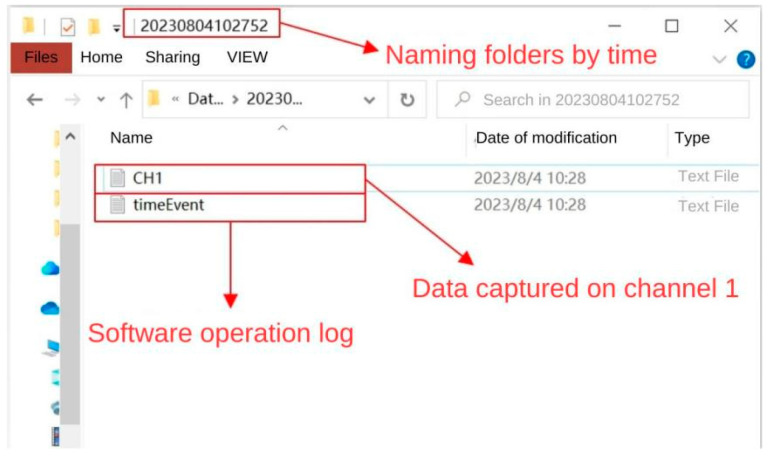
Data Saving for Parachute Cords Tension Acquisition Syst.

During the experimental verification test, the parachute cords tension acquisition system can operate normally, realize data reception and real-time display, draw real-time curves with the collected data, and carry out the parameter calibration and data saving functions, as shown in Figure 33 and Figure 34.

## 5. Experimental Verification of Miniature Measuring Instrument

### 5.1. Experimental Equipment

**Figure 35:**
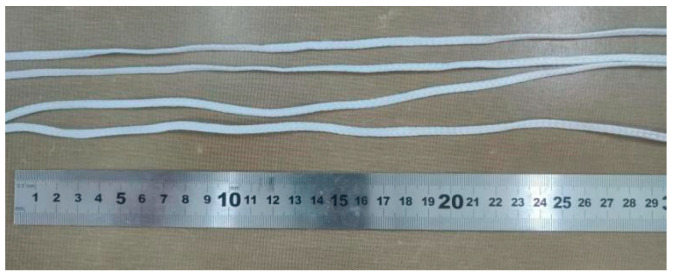
Photo of the Test Parachute Cords.

The hardware equipment required for the experiment includes a computer and a microcomputer-controlled electronic universal testing machine modeled as WDW-50KN, produced by Changchun Jingwang Testing Machine Manufacturing Co., Ltd. The parachute cords used in the experimental testing of the miniature measuring instrument are provided by the Beijing Space Electromechanical Research Institute. Table 8 shows the relevant parameters of the test parachute cords, and Figure 35 shows the test parachute cords.

### 5.2. Experimental Verification of the Prototype of Miniature Measuring Instrument

**Figure 36:**
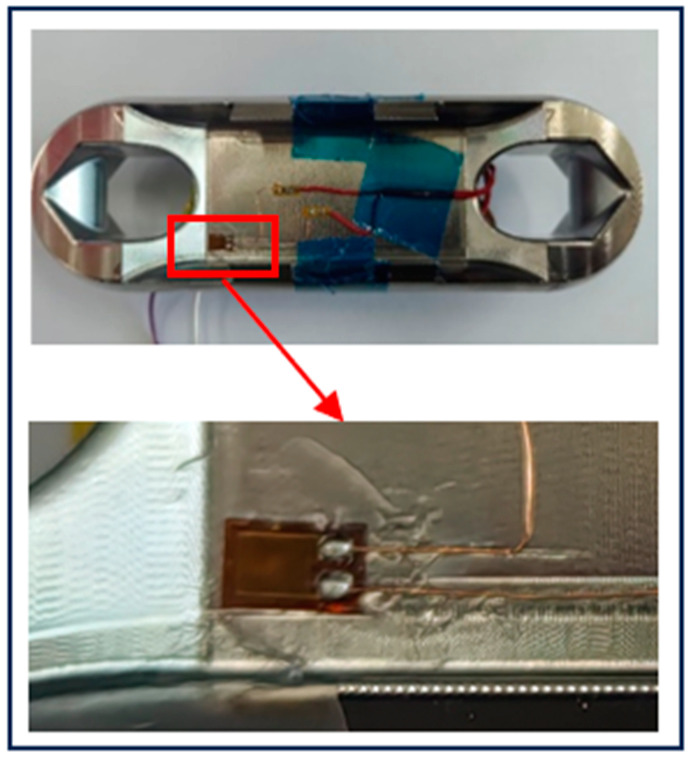
Load Cell I Strain Gage Pasting.

The purpose of this experiment is to verify whether the overall performance of the prototype of the miniature measuring instrument meets the design requirements. Before the start of the test, it is necessary to complete the pasting of the strain gauge which is a single strain gauge and is pasted at the corner of the web with instant adhesive, as shown in Figure 36.

**Figure 37:**
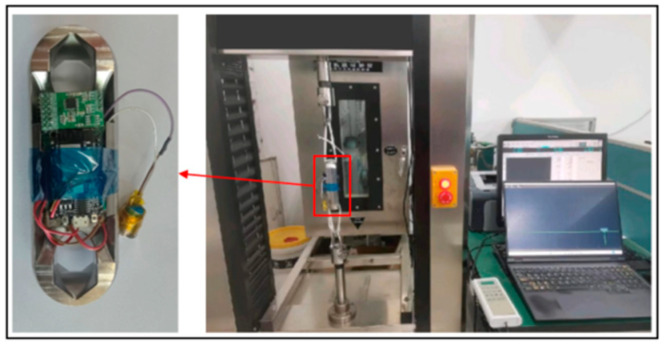
Load Cell I Static Experimental Test.

**Figure 38:**
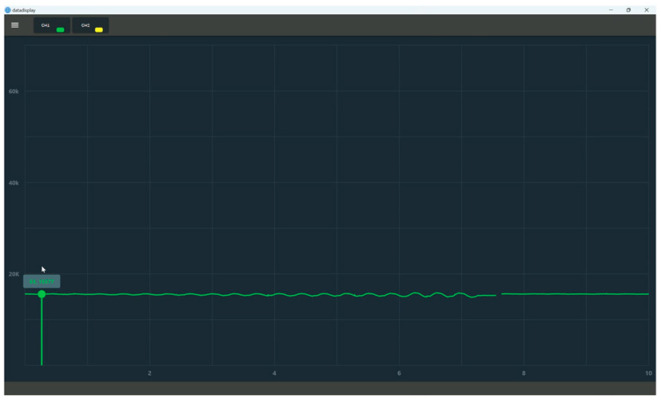
Waveforms Displayed on Upper Computer Software.

The experiment uses a microcomputer-controlled electronic universal testing machine to carry out a force loading test of 0–8000 N on the miniature measuring instrument. The experimental process is shown in Figure 37. The upper computer software displays the force value waveforms in Figure 38 when the load reaches 8000 N. This waveform diagram is used for validating the functionality of the equipment. As shown in the diagram, the equipment is operating normally.

Analysis of the experimental results through experimental testing shows that the prototype of the miniature measuring instrument has the following design defects and fails to meet the design requirements:

1. The size of the acquisition system hardware module I is too large, failing to achieve the design goal of lightweight and miniaturization.
2. The function of the upper computer test software is not perfect, unable to calibrate and save data, and the number of channels is limited to only two. The range of the display interface is large, unable to visualize the experimental results.
3. The Load Cell has the following defects:
  (1) The area that can produce large deformation under force is too small, which makes it difficult to paste the strain gauge.
  (2) The “baffle plate” may rub against the parachute cords in the process of opening, causing damage to the parachute cords.
  (3) The size of the electronic components selected in the hardware selection and design of the acquisition system is large, which leads to the large size of the Load Cell.

### 5.3. Experimental Verification of Load Cell Schemes

**Figure 39:**
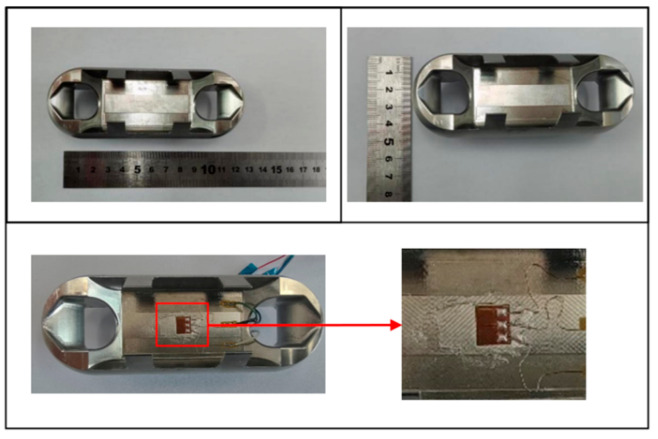
Physical Object and Strain Gage Pasting in Load Cell Scheme II.

**Figure 40:**
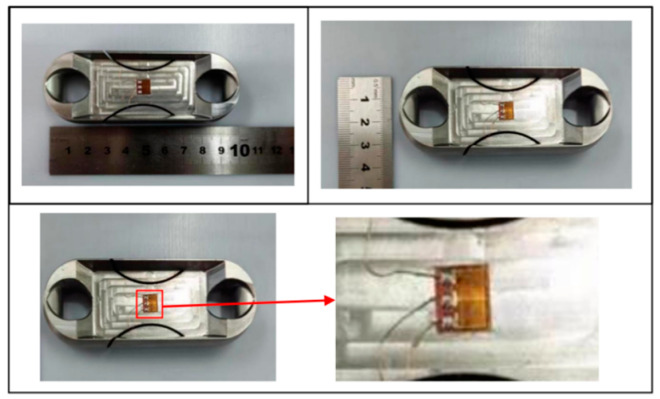
Physical Object and Strain Gage Pasting in Load Cell Scheme III.

**Figure 41:**
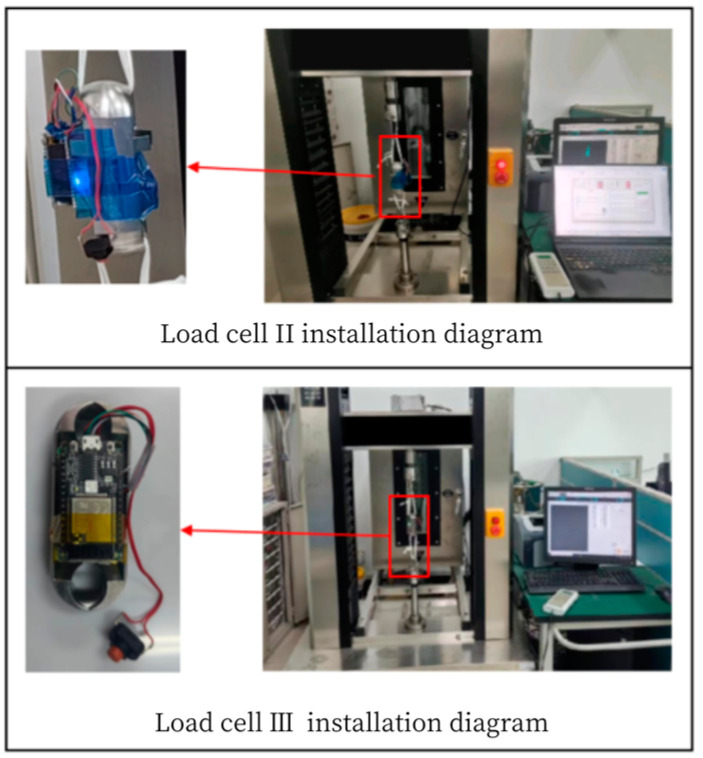
Mounting of the Load Cells to the Universal Testing Machines.

According to the design of Load Cell Schemes II and III, the physical objects are manufactured. A cross line is drawn at the center of the web plate to facilitate the positioning of the strain gages. Figure 39 and Figure 40 show the physical objects and the strain gauge pasted in Load Cell Scheme II and III, respectively. The Load Cells are mounted on the universal testing machines as shown in Figure 41.

**Figure 42:**
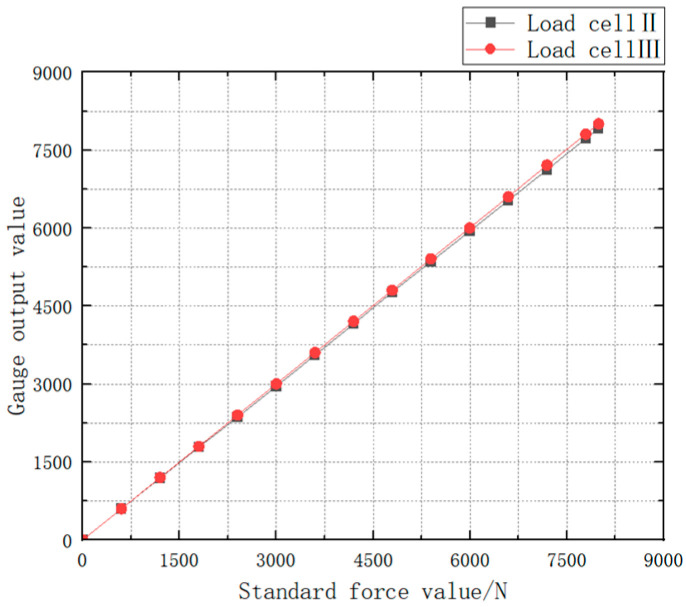
Load Cell Test Data Versus Standard Force Value.

The Load Cell II and the Load Cell III are, respectively, loaded with the force value of 0 ~ 8000 N and the obtained test data are drawn into a curve as shown in Figure 42. The test data of Load Cell I and II is shown in Table 9.

The linearity can be calculated as 1.0256% for Load Cell II and 0.0375% for Load Cell III by using the linearity Formula (7).

(7) $$\gamma_{L}=\pm \frac{{∆L}_{max}}{Y_{FS}}\times 100\%$$

△L_max_ denotes the maximum error value of the static characteristics and the fitted curve.

Meanwhile, the software calibration parameter of Load Cell II in the parachute cords tension acquisition system software is 66,519, and that of Load Cell III is 10,521. Compre-hensively comparing the strain, linearity, and software calibration parameters of Load Cell II and Load Cell III, it can be seen that the sensitivity of Load Cell III is much larger than that of Load Cell II, and the size of Load Cell III is smaller than that of Load Cell II, which meets the demand of the miniature measuring instrument.

## 6. Encapsulation and Calibration of Electronics in High Load Environment

### 6.1. Joint Debugging Test of the Miniature Measuring Instrument

**Figure 43:**
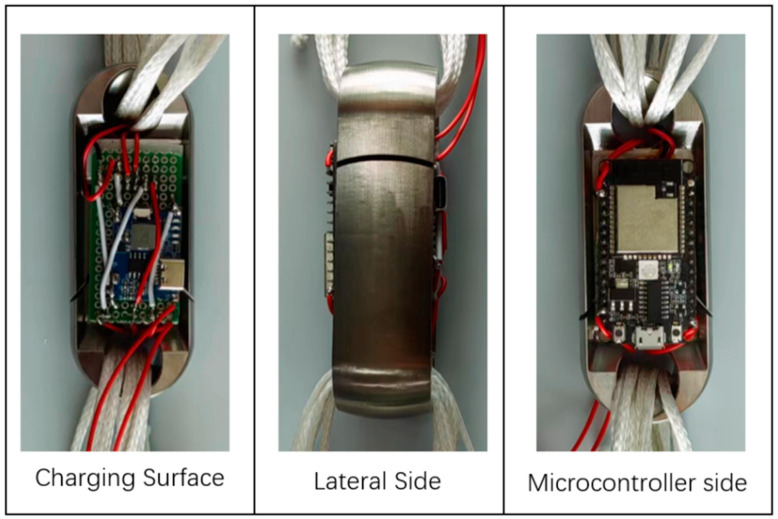
Miniature Measuring Instrument Prototype.

Through the comparison of the Load Cell schemes and the experimental verification test, this paper adopts Load Cell III for the miniature measuring instrument and puts the microcontroller and the battery on one side of the Load Cell, the charging module and the A/D acquisition module on the other side. The modules on both sides are connected through the wires, and the prototype of the miniature measuring instrument after the soldering and assembling is shown in Figure 43.

**Figure 44:**
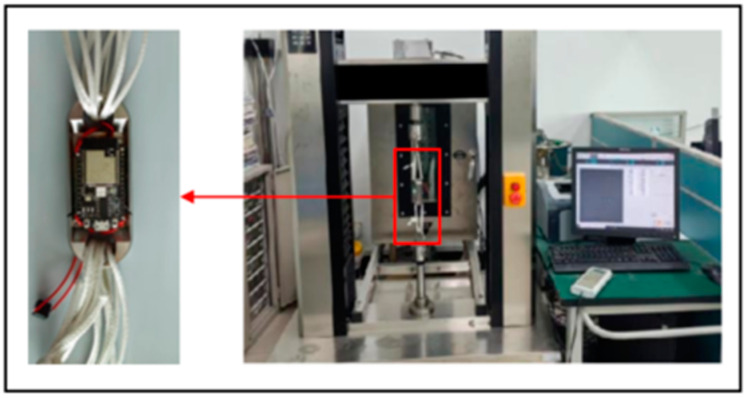
Joint Debugging Experiment.

**Figure 45:**
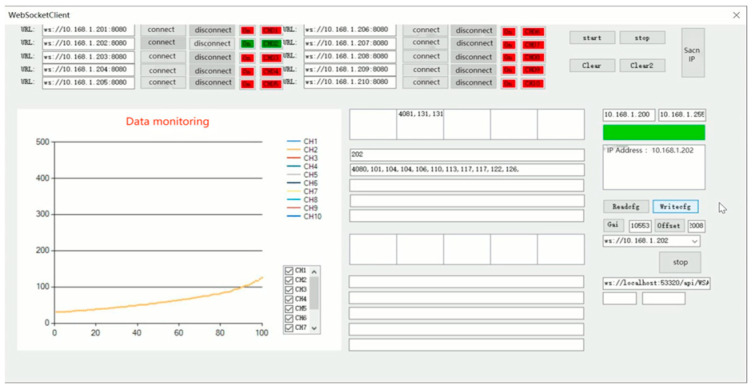
Data Collected by the Parachute Cords Tension Acquisition System.

After completing the hardware connections, testing of the instrument begins. The miniature measuring instrument is connected to the microcomputer-controlled electronic universal testing machine, as shown in Figure 44. Starting from 0 N, stop the universal testing machine action at every 600 N and keep the current force value for 30 s, while the current data of the parachute cords tension acquisition system is recorded. After the universal testing machine is loaded to 8000 N, record the data and finish the testing. The data collected by the parachute cords tension acquisition system is shown in Figure 45.

**Figure 46:**
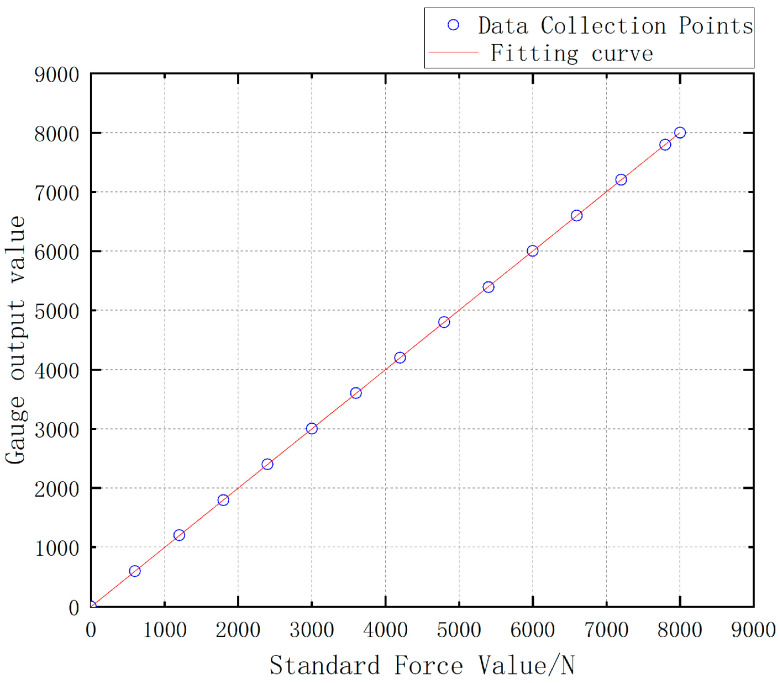
Load Cell AD Sampling Fitting Curve at Static State.

Through the processing of the data in Table 10 and the linear fitting of the curve, it is found that the sample points collected by the miniature measuring instrument experiment are almost in a straight line, as shown in Figure 46, and the fitting result proves that the miniature measuring instrument has better linearity in AD sampling, and according to the above data, the linearity of the structure can be calculated by using Formula (1), which is 0.1124%. During the experiment, the parameter setting of the parachute cords tension acquisition system, “Gai” gain value is 10,521, “offset” bias is 1984.

The joint debugging test experiment proves that the sensitivity and linearity of Load Cell III are better, which meets the needs of the miniature measuring instrument. But before the actual test, it is necessary to encapsulate and protect the strain gauge and electronic components so as to minimize the influence of external factors on the performance of the miniature measuring instrument.

### 6.2. Encapsulation of Miniature Measuring Instrument

**Figure 47:**
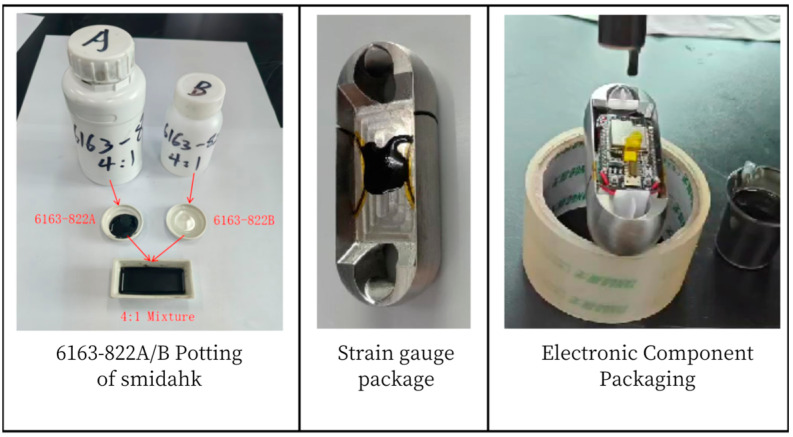
Miniature Measuring Instrument Encapsulating.

**Figure 48:**
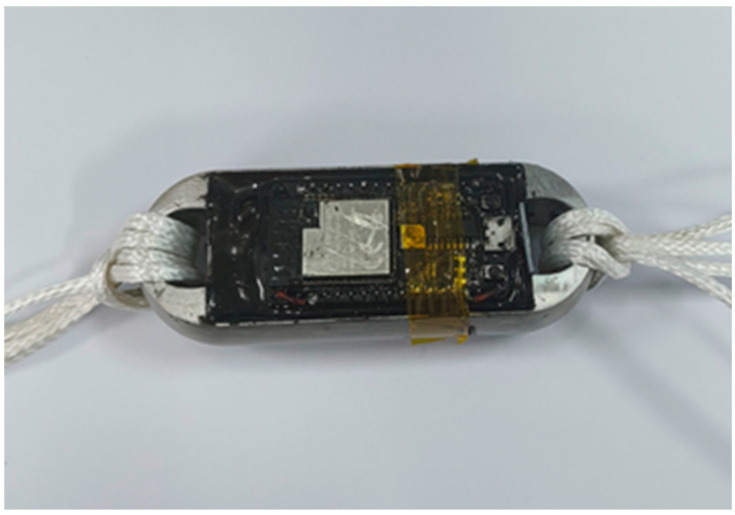
Miniature Measuring Instrument after Encapsulating and Curing.

In order to reduce the impact of external factors on the performance of the miniature measuring instrument, and improve its reliability and durability, 6163-822A/B potting adhesive [18,19,20] is used in the encapsulation. Firstly, encapsulate the strain gauge, and continue to encapsulate the hardware module of the acquisition system of the miniature measuring instrument after the encapsulating adhesive is completely cured, as shown in Figure 47. After the encapsulating adhesive is fully cured (about 36 h), the overall performance of the miniature measuring instrument is tested, and the completed miniature measuring instrument is shown in Figure 48.

### 6.3. Microgage Calibration and Performance Testing

#### 6.3.1. Calibration of the Miniature Measuring Instrument

After completing the encapsulation, it is necessary to calibrate the parameters of the miniature measuring instrument [21] since the working environment after encapsulation is different from that of the bare state, which directly affects its working state and output accuracy. [22]

**Figure 49:**
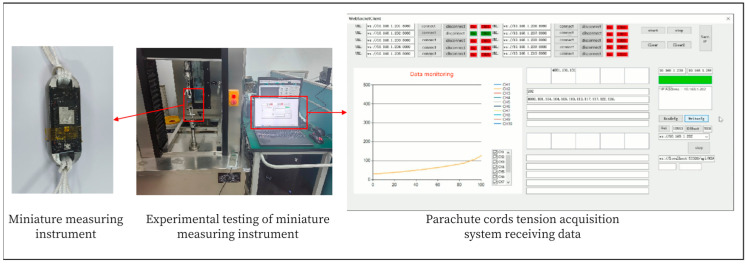
Miniature Measuring Instrument Experimental Testing.

Install the miniature measuring instrument to the universal testing machine, and start the parachute cords tension acquisition system software and the universal testing machine at the same time to begin the experimental testing, as shown in Figure 49.

Use the parameter calibration Formula (8) to calculate the scale factor that needs to be modified, and then modify the Gai value and Offset value for the light and small parachute cords tension instrument to complete the calibration of the parameters.

The modification of the scale factor (C_mod_) can be calculated by using the following formula:

(8) $$C_{mod}=\frac{Standard\;Force\;Value}{Software\;Output\;Value}\times Current\;Scaling\;Factor$$

**Figure 50:**
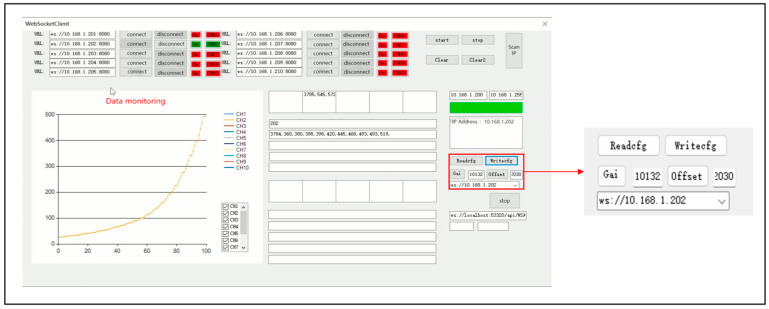
Miniature Measuring Instrument Calibration.

As shown in Figure 50, the parameters of the parachute cords tension acquisition system software after the calibration: “Gai” is 10,132, and “Offset” is 2030.

#### 6.3.2. Miniature Measuring Instrument Performance Testing

In the experiments, the performance parameters of the miniature measuring instrument are obtained by conducting continuous loading tests from 0 to 8000 N on the miniature measuring instrument [23,24,25]:

1. Rated range, 0~8000 N;
2. Resolution of pulling force, 1 N;
3. Repeatability refers to the same input quantity of the sensor for continuity, full range, multiple changes, and the degree of consistency of the static characteristics of the sensor, the basic formula can be expressed as follows:

(9) $$\delta_{R}=\frac{{∆R}_{m}}{Y\left(FS\right)}\times 100\%$$

△Rm indicates the absolute error value of the output quantity corresponding to multiple cyclic changes of the input quantity in the same direction.

**Figure 51:**
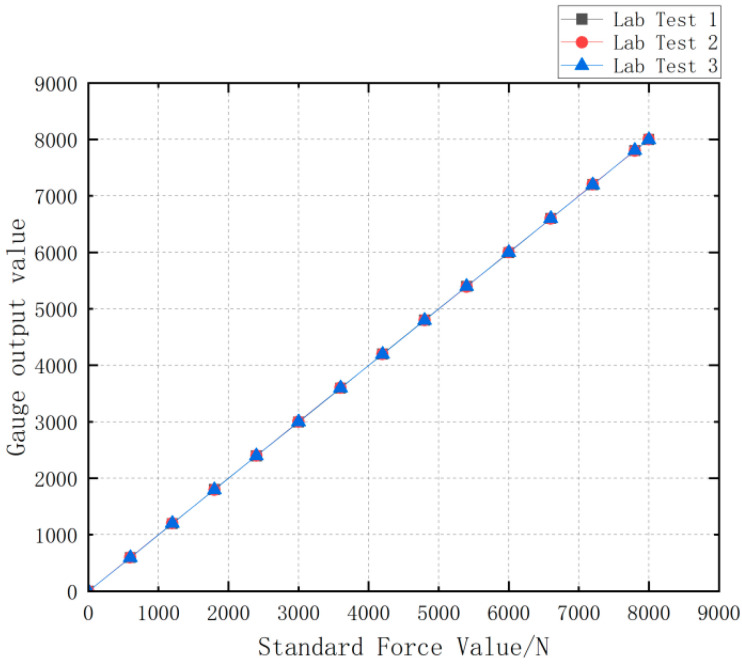
Repeatability Test Curves for Miniature Measuring Instrument.

Three data tests are carried out by gradually increasing the force value, the specific data are shown in Table 11, and the repeatability curve corresponding to the experimental data is shown in Figure 51, with a repeatability of 0.1%.

4. Hysteresis refers to the degree of non-coincidence of the sensor’s positive (input volume increased) and negative (input volume decreased) range characteristics in the rated range. The basic formula can be expressed as follows:

(10) $$\delta_{H}=\frac{\left|{∆H}_{m}\right|}{Y\left(FS\right)}\times 100\%$$

△Hm indicates the maximum error value of positive and negative range output.

**Figure 52:**
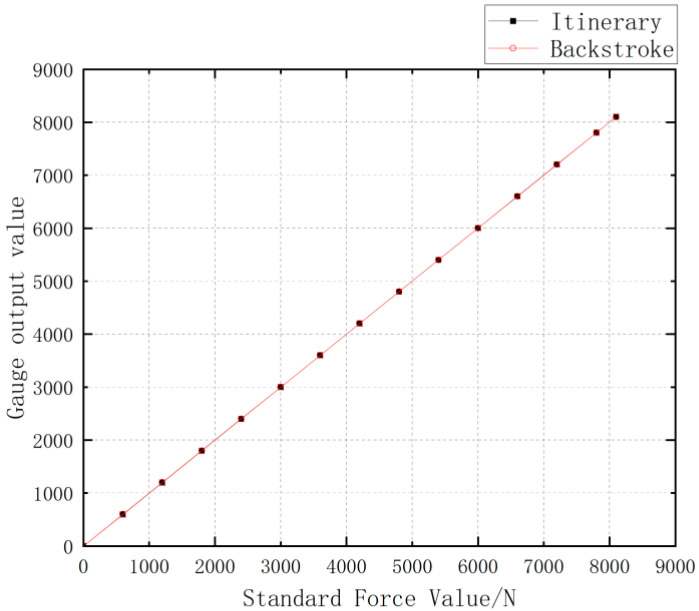
Hysteresis Test Curve for Miniature Measuring Instrument.

Three data tests on the standard force value with gradual increase and decrease are carried out, respectively, and a group of data with the largest deviation in each test is selected, shown in Table 12. The hysteresis test curve is in Figure 52, the test hysteresis result is 0.1%.

5. Sensor input and output quantities from the fitted straight line. Its basic formula can be expressed as.

(11) $$\delta_{L}=\frac{\left|{∆L}_{m}\right|}{Y\left(FS\right)}\times 100\%$$

ΔLm denotes the maximum error value of the static characteristics with respect to the fitted curve.

**Figure 53:**
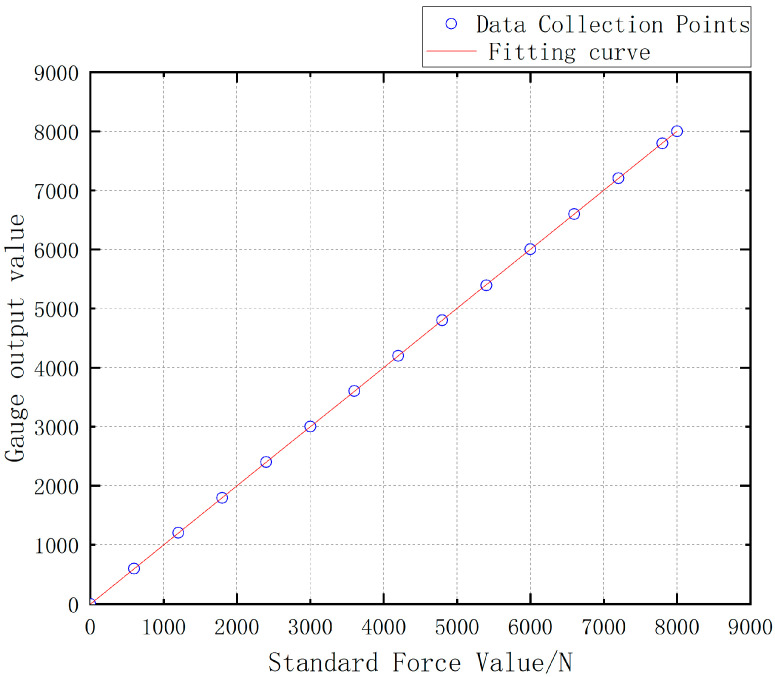
Linearity Test Curve for Miniature Measuring Instrument.

A set of experimental data is detected first and the corresponding fitted data is calculated according to the fitted curve obtained in the previous subsection, as shown in Table 13. The linearity curve of the experimental data is shown in Figure 53, and the linearity of the sensor is tested to be 0.1%.

6. Precision refers to the degree of accuracy of the instrument detection data. According to the hysteresis, repeatability, linearity, and other test results, the measurement accuracy of the miniature measuring instrument is:

(12) $$A=\sqrt{\delta_{R}^{2}+\delta_{H}^{2}+\delta_{L}^{2}}=\sqrt{{0.1\%}^{2}+{0.1\%}^{2}+{0.1\%}^{2}}=0.1732\%$$

7. Sensitivity refers to the output change and the input change ratio of the sensor in the stable operating state. Its basic formula can be expressed as:

(13) $$k=\frac{∆y}{∆x}$$

∆y is the increment of the output quantity and ∆x is the increment of the input quantity. By calculating the data in Table 13 with Equation (13), it can be seen that the sensitivity of the miniature measuring instrument is 1 mV/V.

The specific loading process of the experiment starts from 0 N, increasing in increments of 1000 N to 8000 N, with a total of 8 loading levels. The amplified output voltage values of the miniature measuring instrument were recorded at each level, with the amplification factor set to 128 times.

The measurement experiment data of the miniature measuring instrument is shown in Table 14.

**Figure 54:**
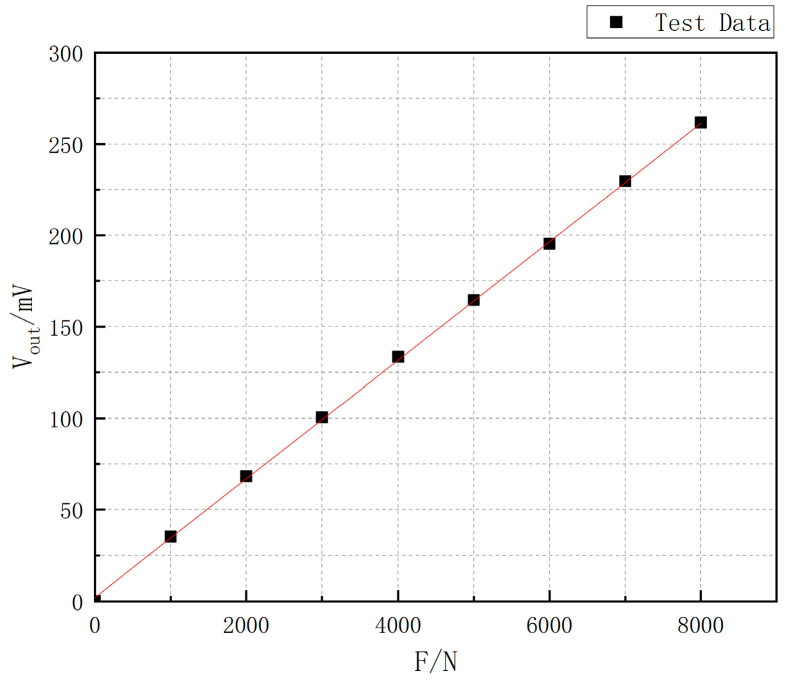
Voltage Output vs. Tensile Force Curve of the Miniature Measuring Instrument.

The voltage output of the micro-measuring instrument corresponding to the tension is shown in Figure 54.

Based on the corresponding curve, the relationship between the voltage output and the tensile force of the miniature measuring instrument is shown in the curve:

(14) $$V_{out}=3.24\times 10^{-2}F+2.07$$

where the output voltage V_out_ is measured in mV and the tensile force F is measured in N. It can also be determined that the relationship between the output voltage of the miniature measuring instrument before amplification and the tensile force of the parachute rope is:

(15) $$V_{out}^{*}=2.53\times 10^{-4}F+1.61\times 10^{-2}$$

By comparing the input-output relationship Formula (15) of the miniature measuring instrument with the theoretical data Formula (6) obtained from Ansys 2022 R1 simulations, it is observed that both are consistent, with errors within an acceptable range. This demonstrates the correctness of the strain gauge model and finite element analysis method proposed in this study, as well as their practical significance.

After the design and manufacturing of the elastomer structure of the miniature measuring instrument and the hardware design and selection of the measurement acquisition system, the assembly and packaging of the miniature measuring instrument were completed. Additionally, the design and development of the parachute rope tensile force acquisition system were completed, along with the coordination testing and calibration of the miniature measuring instrument after packaging. Based on experimental testing, the performance indicators of the miniature measuring instrument were determined, with the main technical parameter values shown in Table 15.

## 7. Summary

The following conclusions are drawn from the study:

1. In this paper, a miniature measuring instrument suitable for measuring the dynamic load of parachute cords has been developed. The instrument which will not affect the structural performance of the parachute can not only provide reliable data for verifying the technical parameters of parachute cords but also provide important support for the future design of stepless deployment of cords.
2. The hardware module of the signal acquisition system with a microcontroller as the core and the corresponding software of the parachute cords tension acquisition system are designed to complete the functions of the acquisition of the dynamic load of the parachute cords, the wireless transmission of the data, the real-time curve plotting and data saving.
3. Based on the hardware module of the acquisition system of the miniature measuring instrument, the design of the Load Cell is completed, and through the static analysis of the Load Cell and the corresponding experimental verification, the final design of the Load Cell and the paste position of the strain gages are determined, which verifies the feasibility of the measurement as well as the reasonableness of the structural design.
4. The research on epoxy potting and stabilizing encapsulation technology has been carried out for electronic instruments under high load environments, and the research on sensor force calibration technology and performance parameter and stability test has been completed by using a microcomputer-controlled electronic universal testing machine, which verifies that the performance of the developed miniature measuring instrument is in line with the expected requirements.

## Figures and Tables

**Table 1:** Expected Indicators for the Miniature Measuring Instrument.

Main Technical Indicators	Expected Parameters
Range	0–8000 N
Sampling Rate	1000 Hz
Measurement Accuracy	3% FS
Measuring Time	>20 min
Weight	<500 g

**Table 2:** Performance Parameters of AI304 Stainless Steel.

Property	Value
Name	AI304 Stainless Steel
Tensile Strength (MPa)	520
Yield Strength (MPa)	215
Elongation (%)	40% (50 mm standard length)
Hardness (HRB)	70
Density (g/cm^3^)	7.93
Thermal Conductivity (W/m·K)	16
Specific Heat Capacity (J/kg·K)	500
Poisson’s Ratio	0.3
Load Cell Modulus (GPa)	193–200
Shear Strength (MPa)	310
Melting Point (°C)	1400–1450

**Table 3:** Axial and Transverse Strain Values of Load Cell III.

Tensile Force Value/N	Axial Strain Value (X-Axis) με	Transverse Strain Value (Y-Axis) με
1000	69.71	−7.65
2000	138.9	−15.25
3000	210.3	−22.99
4000	279.2	−30.13
5000	347.3	−38.9
6000	417.1	−45.49
7000	486.3	−54.05

**Table 4:** Selection of Hardware Module I of the Acquisition System.

Designation	Models
Microcontroller	ESP32C3-32S-Kit Development Board
A/D Sampling Module	ADS8689 Module
Strain Gage Module	Single Bridge Strain Gage Module
Strain Gage	BF350-3AA
Batteries	603040
Battery Charging Module	ETA9742 Module

**Table 5:** Performance Parameters of Signal Acquisition System Hardware Module I.

Technical Indicators	Technical Parameters
Hardware Module Size	67.8 mm × 33.84 mm × 21.63 mm
Weights	56.2 g
A/D Sampling Module Resolution	16-bit
Sampling Rate	100 kHz
Channel Number	8 channels
Supply Voltage	5 V analog power supply, 1.65–5.12 V digital power supply
Battery Capacity	800 mAh

**Table 6:** Signal Acquisition System Hardware Module II.

Designation	Models
Microcontroller	ESP32C3-32S-Kit Development Board
A/D Sampling Module	ADS8689 Module
Strain Gage	BF350-3AA
Batteries Battery Charging Module	603040 ETA9742 Module

**Table 7:** Comparison of Technical Parameters.

Technical Indicators	Signal Acquisition System Hardware Module I	Signal Acquisition System Hardware Module II
Hardware Module Dimensions	67.8 mm × 33.84 mm × 21.63 mm	49.19 mm × 28.22 mm × 22.78 mm
Weight	56.2 g	30 g
A/D Sampling Module Resolution	16 bits	24 bits
Sampling Rate	100 kHz	1000 Hz
Number of Channels	8 channels	Single Channel
Strain gage	1/4 bridge strain gage	Two half-bridge strain gages to form a full bridge
Battery Capacity	800 mAh	400 mAh

**Table 8:** Parameters Related to Parachute Cords.

Material	Width	Thickness	Tensile Strength
Parachute cord	3.5 mm	1 mm	3 KN

**Table 9:** Test Data of Load Cell I and II.

Standard Force Value/N	Load Cell II Test Data	Load Cell III Test Data
0	0	0
599	599	598
1199	1190	1200
1799	1785	1799
2399	2362	2401
2999	2953	3003
3599	3554	3604
4199	4153	4208
4795	4771	4804
5395	5354	5408
5995	5938	6003
6595	6532	6604
7195	7123	7213
7795	7724	7808
7995	7913	8006

**Table 10:** Test Data Received by the Upper Computer.

Standard Force Value/N	Test Data	Fitting Data	Bias Value of the Fit|△Lm|	Percentage of Error%
0	0	0	0	0.00%
599	598	597	1	0.17%
1199	1200	1198	2	0.17%
1799	1799	1798	1	0.06%
2399	2401	2399	2	0.08%
2999	3003	3000	3	0.10%
3599	3604	3600	4	0.11%
4199	4208	4201	7	0.17%
4799	4804	4801	3	0.06%
5399	5408	5402	6	0.11%
5999	6003	6002	1	0.02%
6599	6604	6603	1	0.02%
7199	7213	7204	9	0.13%
7799	7808	7804	4	0.05%
8000	8006	8005	1	0.01%

**Table 11:** Repeatability Test Experimental Data for Miniature Measuring Instrument (Gradual Increase in Pulling Force).

Standard Force Value/N	S1	S2	S3	Maximum Error △ Rmax	Percentage of Error%
0	0	0	0	0	0.00%
599	596	598	597	3	0.50%
1199	1197	1202	1200	3	0.25%
1799	1800	1795	1797	5	0.28%
2399	2397	2399	2398	2	0.08%
2999	2995	3003	2997	4	0.13%
3599	3597	3602	3599	3	0.08%
4199	4200	4197	4198	4	0.10%
4799	4800	4799	4797	3	0.06%
5399	5400	5391	5397	8	0.15%
5999	5995	6002	6000	4	0.07%
6599	6600	6597	6599	3	0.05%
7199	7200	7204	7197	5	0.07%
7799	7803	7797	7807	8	0.10%
7999	8000	8002	7996	8	0.10%

**Table 12:** Experimental Data for Hysteresis Test of Miniature Measuring Instrument.

Standard Force Value/N	Positive Range Test Data	Reverse Range Test Data	Difference △Hm	Percentage of Error%
0	0	0	0	0%
599	600	601	1	0.17%
1199	1200	1202	2	0.17%
1799	1798	1801	3	0.17%
2399	2402	2403	4	0.17%
2999	3004	3007	7	0.23%
3599	3602	3604	4	0.11%
4199	4201	4202	1	0.02%
4799	4802	4803	2	0.04%
5399	5403	5405	5	0.09%
5999	6005	6006	6	0.10%
6599	6604	6606	3	0.05%
7199	7204	7205	2	0.03%
7799	7804	7805	2	0.03%
8099	8104	8105	2	0.02%

**Table 13:** Experimental Data for Linearity Test of Miniature Measuring Instrument.

Standard Force Value/N	Test Data	Fitting Data	Bias Value of the Fit|△Lm|	Percentage of Error%
0	0	0	0	0.00%
599	598	597	1	0.17%
1199	1200	1198	2	0.17%
1799	1799	1798	1	0.06%
2399	2401	2399	2	0.08%
2999	3003	3000	3	0.10%
3599	3604	3600	4	0.11%
4199	4205	4201	4	0.10%
4799	4804	4801	3	0.06%
5399	5405	5402	3	0.06%
5999	6003	6002	1	0.02%
6599	6604	6603	1	0.02%
7199	7209	7204	5	0.07%
7799	7808	7804	4	0.05%
8000	8006	8005	1	0.01%

**Table 14:** Voltage Output Data of the Miniature Measuring Instrument.

Tensile Force/N	Miniature Measuring Instrument Output Voltage/mV
1000	35.2
2000	68.3
3000	100.5
4000	133.5
5000	164.6
6000	195.4
7000	229.6
8000	261.8

**Table 15:** Miniature Measuring Instrument Performance Indicators.

Performance Indicators	Technical Parameters
Instrument Dimensions	96 mm × 36 mm × 29.5 mm (L × W × H)
Weight	291 g
Endurance	>20 min
Range	0~8000 N
Resolution	1 N
Sensitivity	1 mV/V
Repeatability	0.1%
Hysteresis	0.1%
Linearity	0.1%
Accuracy	0.1732%

## Data Availability

Data are contained within the article.
